# Structure, catalysis, and inhibition mechanism of prenyltransferase

**DOI:** 10.1002/iub.2418

**Published:** 2020-11-27

**Authors:** Hsin‐Yang Chang, Tien‐Hsing Cheng, Andrew H.‐J. Wang

**Affiliations:** ^1^ Department of Life Sciences and Institute of Genome Sciences National Yang‐Ming University Taipei Taiwan; ^2^ Institute of Biological Chemistry Academia Sinica Taipei Taiwan

**Keywords:** farnesyl diphosphate, isoprenoid, prenyltransferase, terpene, terpenoid

## Abstract

Isoprenoids, also known as terpenes or terpenoids, represent a large family of natural products composed of five‐carbon isopentenyl diphosphate or its isomer dimethylallyl diphosphate as the building blocks. Isoprenoids are structurally and functionally diverse and include dolichols, steroid hormones, carotenoids, retinoids, aromatic metabolites, the isoprenoid side‐chain of ubiquinone, and isoprenoid attached signaling proteins. Productions of isoprenoids are catalyzed by a group of enzymes known as prenyltransferases, such as farnesyltransferases, geranylgeranyltransferases, terpenoid cyclase, squalene synthase, aromatic prenyltransferase, and *cis‐* and *trans‐*prenyltransferases. Because these enzymes are key in cellular processes and metabolic pathways, they are expected to be potential targets in new drug discovery. In this review, six distinct subsets of characterized prenyltransferases are structurally and mechanistically classified, including (1) head‐to‐tail prenyl synthase, (2) head‐to‐head prenyl synthase, (3) head‐to‐middle prenyl synthase, (4) terpenoid cyclase, (5) aromatic prenyltransferase, and (6) protein prenylation. Inhibitors of those enzymes for potential therapies against several diseases are discussed. Lastly, recent results on the structures of integral membrane enzyme, undecaprenyl pyrophosphate phosphatase, are also discussed.

**Abbreviations**
ABBA: α‐β‐β‐α barrel
AtPPPS: polyprenyl pyrophosphate synthase in Arabidopsis
BacA/UPPP: undecaprenyl pyrophosphate phosphatase
CLPP: cyclolavandulyl diphosphate
CLPPS: cyclolavandulyl diphosphate synthase
DHDDS: dehydrodolichyl diphosphate synthase
DHS: dehydrosqualene
DMAPP: dimethylallyl diphosphate
DMATS: dimethylallyltryptophan synthase
DolP: dolichol phosphate
FPG‐trisaccharide: cis‐farnesyl group in the phosphoglycolipid
FPP: farnesyl diphosphate
FPPS: farnesyl diphosphate synthase
FsPP: farnesyl thiopyrophosphate
FTase: farnesyltransferase
GPP: geranyl diphosphate
GGPP: geranylgeranyl diphosphate
GGPPS: geranylgeranyl diphosphate synthase
GGTase: geranylgeranyl transferase
GLPP: geranyl lavandulyl diphosphate
GPPS: geranyl diphosphate synthase
HepS and HspT: heptaprenyl diphosphate synthase
HexPPS: trans‐hexaprenyl diphosphate synthase
HSQ: hydroxysqualene
IPP: isopentenyl diphosphate
ISLPP: isosesquilavandulyl diphosphate
LPP: lavandulyl diphosphate
LPPS: lavandulyl diphosphate synthase
LSU: large subunit
Mcl22: isosesquilavandulyl diphosphate synthase
MEP: methylerythritol phosphate
MVA: mevalonate
OPP: octaprenyl diphosphate
OPPS: octaprenyl pyrophosphate synthase
PSPP: presqualene diphosphate
SqhC: tetraprenyl‐β‐curcumene cyclase
SQS: squalene synthase
SSU: small subunit
UP: undecaprenyl phosphate
UPP: undecaprenyl pyrophosphate
UPPS: undecaprenyl diphosphate synthase
YtpB: tetraprenyl‐β‐curcumene synthase
Z,E‐DecPP: decaprenyl diphosphate
zFPS: Z,Z‐farnesyl diphosphate synthase

## 1 INTRODUCTION

Isoprenoids, also known as terpenes or terpenoids, are a group of natural products diverse in structure and biological function. To date, more than 80,000 identified members have been characterized structurally and chemically. They are widely distributed in living organisms, including microbes, insects, plants, and marine organisms.^1^ For instance, carotenoids and retinoids are involved in light‐sensitive elements that play a role in light absorption in plants.^2^ Steroid hormones derived from cholesterol play a central role in lipid physiology in the human body.^3^, ^4^ Ubiquinone and menaquinone are lipophilic metabolites functioning as electron carriers in the respiratory chain in prokaryotes and eukaryotes.^5^ Undecaprenyl pyrophosphate (UPP) serves as a membrane‐associated glycan carrier in the bacterial cell wall synthesis.^6^, ^7^ Dolichol plays a role in glycoprotein biosynthesis and posttranslational modifications in the endoplasmic reticulum (ER).^8^

**SCHEME 1:**
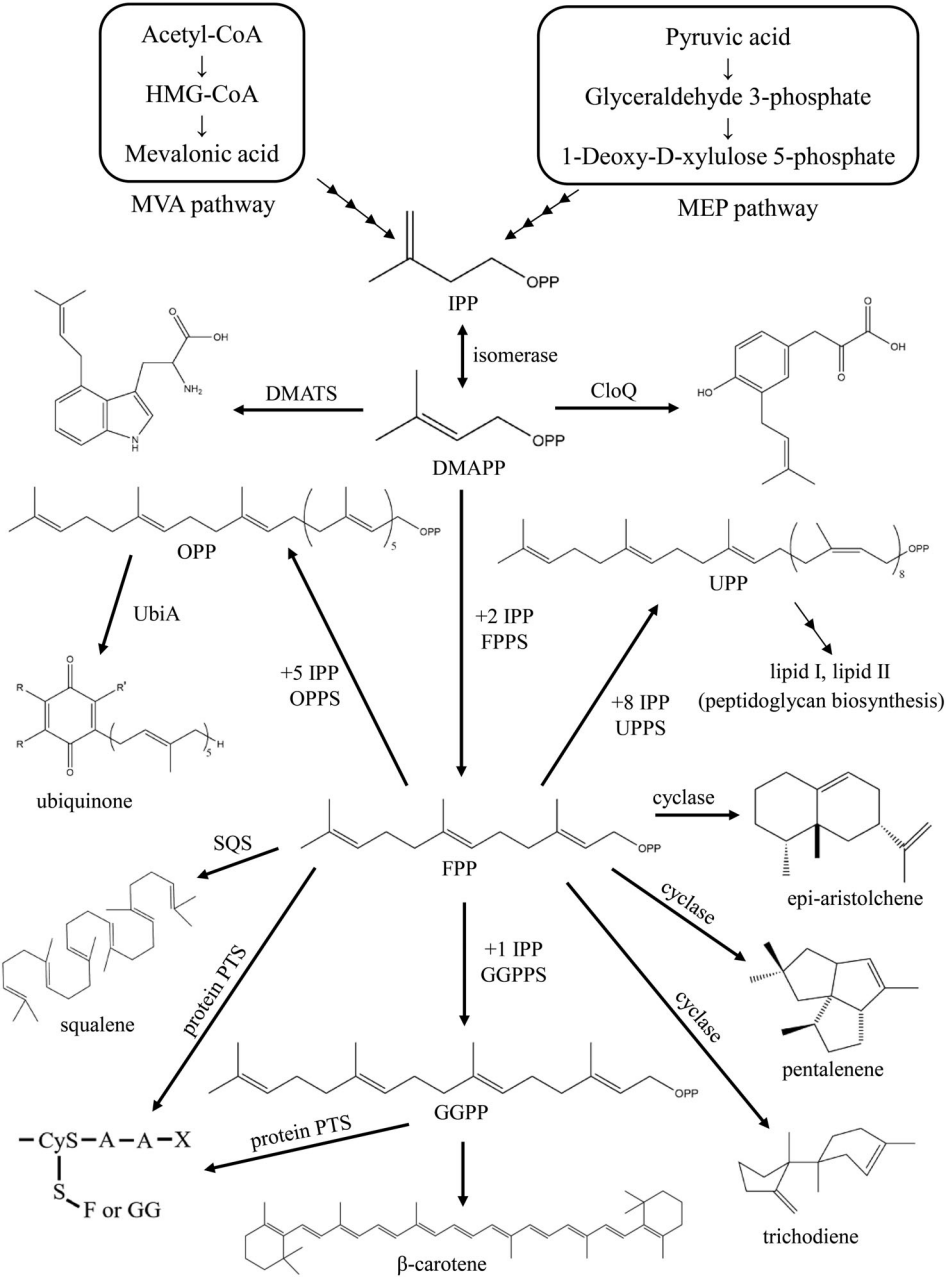
Prenyltransferases can condense with isopentenyl diphosphate and dimethylallyl diphosphate to form various natural isoprenoids with different chain lengths

All these isoprenoid compounds are derived from linear prenyl diphosphate molecules, which are composed of two 5‐carbon intermediates, isopentenyl diphosphate (IPP) and its isomer dimethylallyl diphosphate (DMAPP) as the building blocks. Both IPP and DMAPP are synthesized via two distinct pathways: the mevalonate (MVA) and methylerythritol phosphate (MEP) pathways (Scheme 1). The MVA pathway is observed in animals, fungi, archaea, and some bacteria, and the synthesis of isoprene units in liver tissues and yeast through this pathway was first investigated in the 1950s.^9^ By contrast, most bacteria synthesize isoprene through the MEP pathway.^10^ A group of enzymes called prenyltransferases can subsequently condense with IPP and DMAPP to form various isoprenoid diphosphates with different chain lengths, such as 10‐carbon geranyl diphosphate (GPP), 15‐carbon farnesyl diphosphate (FPP), and 20‐carbon geranylgeranyl diphosphate (GGPP), catalyzed by GPP synthase, FPP synthase and GGPP synthase, respectively (Scheme 1).^11^, ^12^, ^13^ The carbon chain lengths of linear isoprenoids widely range from 10‐carbon GPP to natural rubber (C_>10,000_).^14^

All carbon skeletons are generated from linear isoprenoids (e.g., GPP, FPP, and GGPP) through consecutive condensation reactions with IPP or DMAPP in the biosynthesis of isoprenoids. These 10–20 carbon linear isoprenoid compounds serve as starting materials of prenyltransferases for the biosynthesis of a wide variety of isoprenoid natural products. For example, FPP can react with IPP through a so‐called head‐to‐tail condensation reaction to form a 20‐carbon GGPP as a building block in plant carotenoid biosynthesis.^15^, ^16^ Two FPP molecules are catalyzed through two‐step reductive head‐to‐head condensation by squalene synthase (SQS) to form squalene, a precursor in cholesterol and steroid hormone synthesis in plants and animals.^17^, ^18^ One GPP molecule and the *cis*‐farnesyl group in phosphoglycolipid (FPG‐ trisaccharide) are catalyzed by a unique head‐to‐middle prenyltransferase, MoeN5, to produce the 25‐carbon moenocinyl side‐chain–containing lipid in moenomycin biosynthesis.^19^, ^20^ Terpenoid cyclase catalyzes FPP cyclization to form the cyclic sesquiterpene hydrocarbon trichodiene, a precursor in antibiotic and mycotoxin biosynthesis.^21^ Two aromatic prenyltransferase, namely CloQ and NphB, can catalyze the transfer of a 5‐carbon DMAPP or 10‐carbon GPP onto electron‐rich aromatic acceptor molecules (Scheme 1).^22^, ^23^ These natural products are primary and secondary metabolites of living organisms and are important in pharmaceutical research. Either FPP or GGPP can be attached through covalent binding to a conserved cysteine of certain signaling proteins, such as Ras‐family GTPases, via an irreversible posttranslational modification.^24^, ^25^ Prenylation of Ras‐related GTP‐binding proteins is essential for the proper cellular activity in signal transduction pathways.^26^, ^27^

In this review, we summarized previous studies on and recent research progresses in enzymatic structures, catalytic mechanisms, and biological functions of prenyltransferase in living organisms. We suggested that a diverse range of prenyltransferase can be classified into six main classes on the basis of their distinct catalytic mechanisms: (1) head‐to‐tail, (2) head‐to‐head, (3) head‐to‐middle condensation, (4) terpenoid cyclase, (5) aromatic prenyltransferase, and (6) protein prenylation. Potential prenyltransferase inhibitors for novel therapies against several diseases are also discussed. A list of enzymes and isoprenoids discussed in this review and their abbreviations is provided in Table S1.

## 2 CLASS 1: HEAD‐TO‐TAIL PRENYL SYNTHASE

### 2.1 Z (*cis*)‐ and E (*trans*)‐type prenyltransferases

Isoprenoids have diverse structures derived from the coupling of allylic substrates, such as IPP, DMAPP, GPP, FPP, and GGPP. These linear isoprenyl diphosphates are catalyzed through a canonical consecutive head‐to‐tail condensation reaction by prenlytransferases (or prenyl synthase), followed by various modifications, such as transformation, cyclization, and glycosylation, in the biosynthesis of molecules including steroids, carotenoids,^2^, ^3^ the side chains of respiratory quinones,^5^ natural rubber,^14^ and glycosyl carrier lipid^6^, ^7^ (Scheme 1).

**FIGURE 1:**
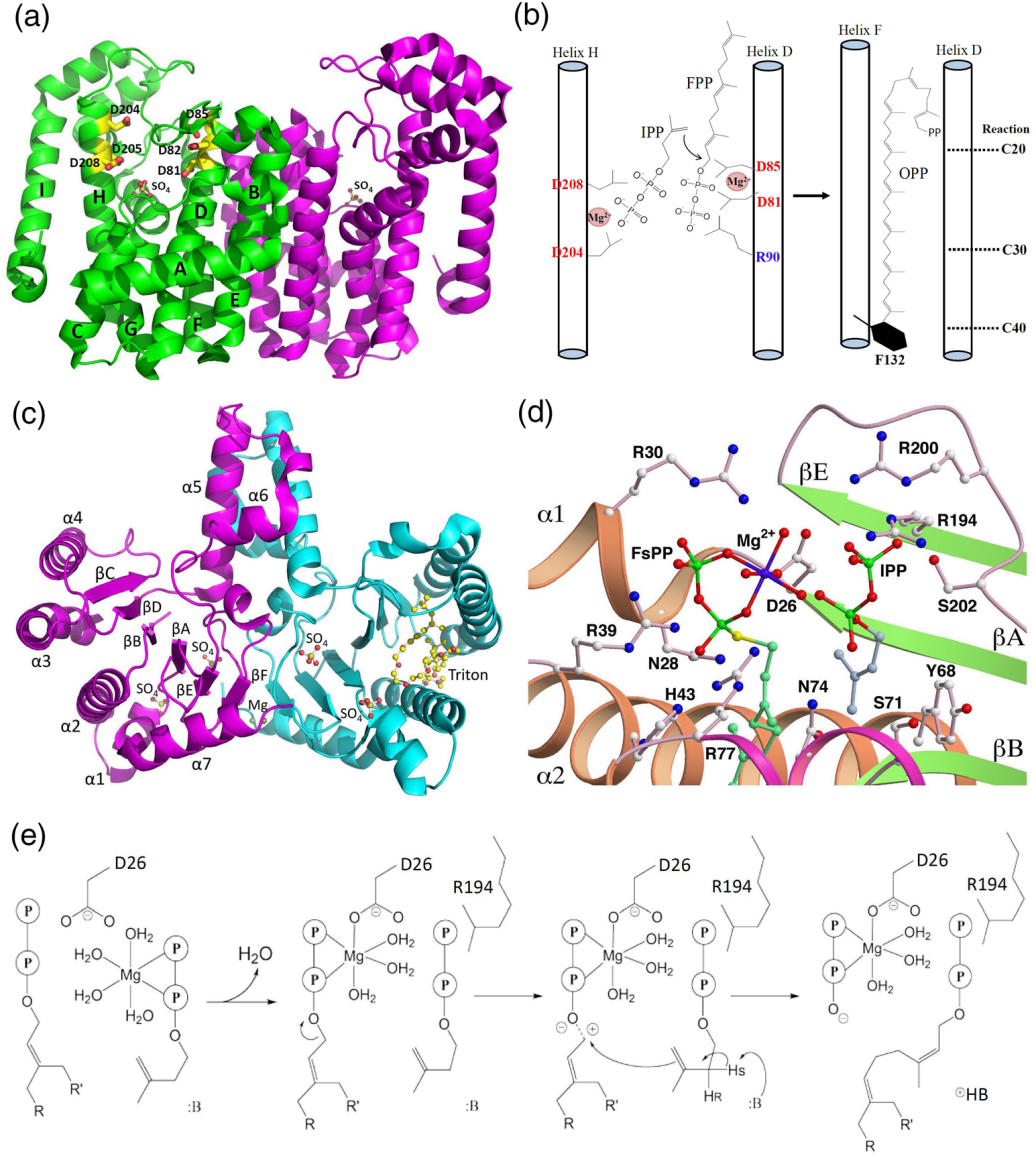
Overall structures of *Thermotoga maritima* octaprenyl pyrophosphate synthase (OPPS) and *Escherichia coli* undecaprenyl diphosphate synthase (UPPS). (a) Two identical subunits of OPPS (299 residues) dimerize (green and magenta), and two sulfate ions are in the active site from each subunit. (b) Proposed reaction mechanism and chain length upon OPPS‐mediated catalysis. The bulky hydrophobic residue Phe‐132 located at the bottom of helix F potentially inhibits further chain elongation of OPP and determines the final carbon chain length. (c) UPPS dimerizes (magenta and cyan), and each subunit comprises 253 residues. In the crystal structure, UPPS is in complex with sulfate and magnesium ions and Triton X‐100 molecules, and each monomer has two sulfate groups (termed S1 and S2) bound in the active site. (d) Structural model of UPPS bound with FsPP (FPP analogue) at the S1site and IPP in the S2 site based on the crystal structures of WTF (UPPS + FsPP + IPP) and MTI (D26A mutant + IPP).^32^ Reprinted from our previously published data (Reference 32). Originally published in Reference 32. Copyright 2005 American Society for Biochemistry & Molecular Biology. (e) The plausible mechanism of the UPPS reaction

On the basis of the stereochemistry of the double bond formation, prenlytransferases are classified as Z and E types, which catalyze the formation of *cis* and *trans* double bonds, respectively, through condensation with specific numbers of IPP.^28^, ^29^ *Trans*‐prenyltransferases typically share two conserved aspartate‐rich DDXXD (or DXXXD) motifs facing each other on opposite helices of the substrate binding pocket and tend to synthesize short‐ and medium‐chain‐length products ranging from 15 to 50 carbon atoms.^30^, ^31^ For instance, in octaprenyl pyrophosphate synthase (OPPS), the first aspartate‐rich motif (S1 site) binds with FPP, whereas the second motif (S2 site) binds IPP with Mg^2+^ ion. The C3–C4 double bond of IPP subsequently attacks FPP at C1 through five consecutive head‐to‐tail condensation reactions to synthesize 40‐carbon long‐chain OPP (Figure 1a,b).^33^ This polymer serves as the side‐chain of ubiquinone (or menaquinone) and mediates electron transfer in the respiratory chain.^34^ Other well‐known *trans*‐type enzymes, such as farnesyl diphosphate synthase (FPPS) and geranylgeranyl diphosphate synthase (GGPPS), have been reported to synthesize 15‐carbon FPP and 20‐carbon GGPP, respectively.^12^, ^13^ Unlike *trans*‐prenyltransferases, *cis*‐prenyltransferases do not contain the DDXXD motif and mostly generate ≥C55 long‐chain polymers. A well‐known archetypal example is undecaprenyl diphosphate synthase (UPPS). During the catalysis of head‐to‐tail condensation, FPP binds with Mg^2+^ ion in the S1 site and with IPP in the S2 site to yield 55‐carbon undecaprenyl diphosphate (UPP), which serves as a lipid carrier in bacterial cell wall biosynthesis (Figure 1d–e).^32^, ^35^, ^36^, ^37^, ^38^, ^39^, ^40^ The *cis‐* and *trans‐*prenyltransferases do not share similarity in both primary and tertiary structures, despite the similar catalytic mechanisms.

### 2.2 Homodimeric and heteromeric prenyltransferase

Prenyltransferases are classified as *trans‐* and *cis*‐prenyltransferases. On the basis of the protein composition, *trans*‐prenyltransferases can be further divided into homodimeric and hetero‐oligomeric enzymes. Over the past decades, the core machinery for *trans*‐homodimeric enzymes has been identified clearly, and the structural information of several crucial enzyme–substrate complexes has been proposed. In general, two identical subunits are associated to form a dimer with a twofold axis at the interface, and each subunit contains a deep hydrophobic cavity surrounded by helices for the factor and allylic substrate binding, which form longer chain products. The relevant examples include FPPS (15‐carbon), GGPPS (20‐carbon), and OPPS (40‐carbon).^12^, ^13^, ^34^ Notably, Hsieh et al. conducted a series of crystal structures and genetic complementation analyses and proposed that a novel homodimeric polyprenyl pyrophosphate synthase in *Arabidopsis* (AtPPPS) that can synthesize multiple 25‐carbon (medium‐chain) to 45‐carbon (long‐chain) products. This finding is consistent with the mutagenesis data (I99F/V162F), which concluded that the hydrophobic tunnel can accommodate products >10‐carbon GPP.^41^ Therefore, the authors suggested that in *Arabidopsis*, the precursor GPP for 10‐carbon monoterpene biosynthesis is produced by heteromeric AtPPPS (AtGPPS),^42^ but not homomeric AtPPPS.^41^

**FIGURE 2:**
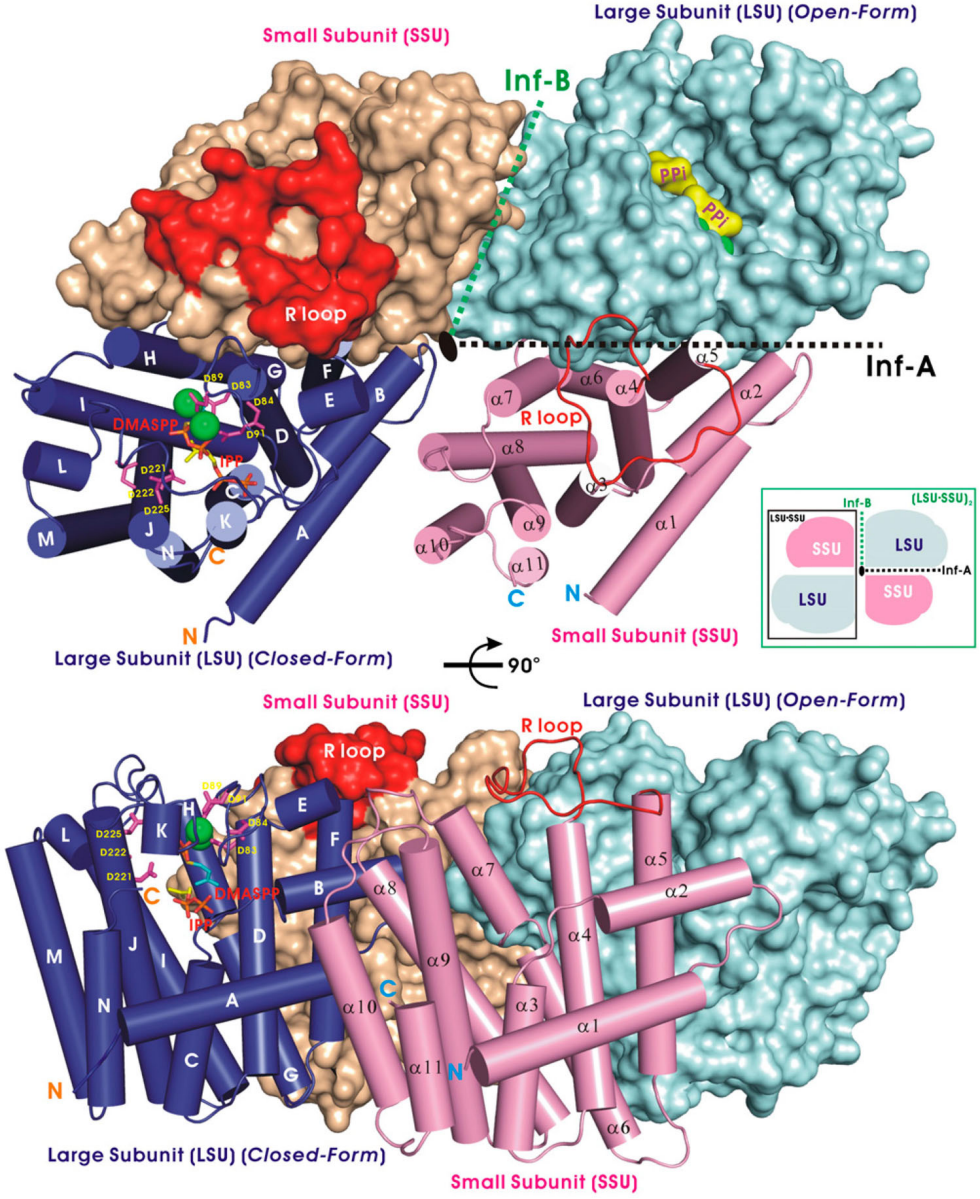
Crystal structure of heterotetrameric geranylgeranyl diphosphate synthase (LSU·SSU)_2_ from *Mentha piperita*. LSU subunits are presented as cylinders (blue) and a filled surface (cyan), and SSU subunits are displayed as cylinders (magenta) and a surface model (wheat‐colored). An LSU subunit, a catalytic unit, bound with DMASPP (DMAPP analog) and IPP molecules are shown as sticks and Mg^2+^ ions (green balls) in the crystal structure. The orientation of the bottom panel is rotated 90° around the horizontal axis relative to the top surface. Reprinted from our previously published data (Reference 43). Originally published in Reference 43. Copyright 2010 American Society of Plant Biologists

The catalytic mechanism and structural information of hetero‐oligomeric prenyltransferase was not well understood until 2010, when Chang et al. proposed the crystal structure of *trans*‐heterotetrameric GPPS [(LSU·SSU)_2_‐type, where LSU = large subunit and SSU = small subunit] extracted from mint (*Mentha piperita*) and demonstrated that it was involved in menthol biosynthesis in mint glandular trichomes (Figure 2).^43^ LSU has high sequence similarity (>50% identity) with homodimeric prenyltransferases as a catalytic subunit, while SSU has less sequence similarity (~15% identity) with other enzymes and lacks the DDXXD (or DXXXD) motif as a noncatalytic subunit. Biochemical and structural analyses have suggested that the LSU serves as a catalytic unit, whereas the SSU may act as a regulatory unit. No activity was detected without the presence of either, probably because the monomer cannot fold a stable structure for enzyme function.^43^ Within a similar time frame, Sasaki et al. reported the crystal structure of heterodimeric *trans*‐hexaprenyl diphosphate synthase (HexPPS) from *Micrococcus luteus* B‐P 26. This enzyme is composed of an LSU (HexB) and an SSU (HexA) and catalyzes three consecutive condensations of IPP on FPP to produce HexPP (30 carbons). HexB contains two aspartate‐rich motifs responsible for catalysis in substrate condensation, whereas HexA may control the product chain length by using the size of the hydrophobic cavity as a molecular ruler in cooperation with HexB.^44^ Another functionally known *trans*‐heterodimeric enzyme is heptaprenyl diphosphate synthase (HepS and HspT), which catalyzes one FPP and four IPP molecules through a head‐to‐tail condensation reaction to form a 35‐carbon heptaprenyl diphosphate in *Bacillus subtilis*.^45^ However, its structural information remains unclear (see Section 5.2. New subclass: Sesquarterpene cyclase for details).

Grabinska et al. also classified *cis‐*prenyltransferase into two subfamilies of homodimeric and heteromeric enzymes.^14^ The homodimeric *cis‐*prenyltransferases are found in bacteria and plants and generally produce short‐ and medium‐chain (10–55‐carbon) prenols, including *Z,E*‐FPP from *Mycobacterium tuberculosis* (Rv1086),^46^ nerylneryl diphosphate (20‐carbon) from *Solanum* sp.,^47^ UPP from various bacteria,^6^, ^7^ and decaprenyl diphosphate (*Z,E*‐DecPP, 50‐carbon) from *M. tuberculosis*.^48^ The biological function of *Z,E*‐DecPP is somewhat similar to that of bacterial UPP, which is essential in bacterial cell wall biosynthesis. By contrast, the heteromeric *cis‐*prenyltransferases comprise catalytic and noncatalytic subunits and generally synthesize long‐chain (>70‐carbon) prenols.^14^ These heteromeric enzymes, such as dehydrodolichyl diphosphate synthase (DHDDS), are typically found in fungi, metazoan, plants, and animals.^14^, ^49^ Their catalytic subunits have a high similarity with UPPS‐like enzymes, whereas their noncatalytic subunits exhibit sequence similarity with other known *cis‐*prenyltransferases only in the C‐terminus. In eukaryotic cells, DHDDS plays an essential role in the synthesis of dolichol phosphate (DolP; 55–100‐carbon long), a lipid carrier necessary for protein glycosylation reactions in the ER.^50^ Although the physiological functions of several DHDDS enzymes have been investigated for more than a decade, including NgBR/hCIT in mammals,^51^ Nus1/Rer2 or Nus1/Srt1 in yeasts,^50^ SpNus1/SpRer2 in fungi,^51^ SlCPT3/SlCPTBP in tomato plants,^52^ and Lew1/At2g17570 in *Arabidopsis* ^53^; to date, no structure of a heteromeric DHDDS is available. A breakthrough was achieved when Ma et al. recently reported the first crystal structure of Nus1, the noncatalytic subunit of DHDDS from *Saccharomyces cerevisiae*.^50^ The structural modeling of the Nus1/Rer2 heterodimer suggested that the C‐terminus of Nus1 participates in substrate binding through Asn372, a C‐terminal residue. Its equivalent residue in human NgBR and most other *cis‐*prenyltransferases is arginine in the C‐terminal conserved RXG motif, involved in IPP binding.^50^

To date, the core machinery and structural information on several crucial enzyme–substrate complexes of homodimeric *cis‐*prenyltransferases have been clearly identified, but the molecular machinery responsible for isoprenoid biosynthesis in heteromeric *cis‐*prenyltransferases is relatively unclear. Therefore, additional studies on the molecular structures, mechanisms, and biological functions of heterodimeric or heteromeric *cis‐*prenyltransferase are warranted.

### 2.3 Prenyltransferase inhibition

**FIGURE 3:**
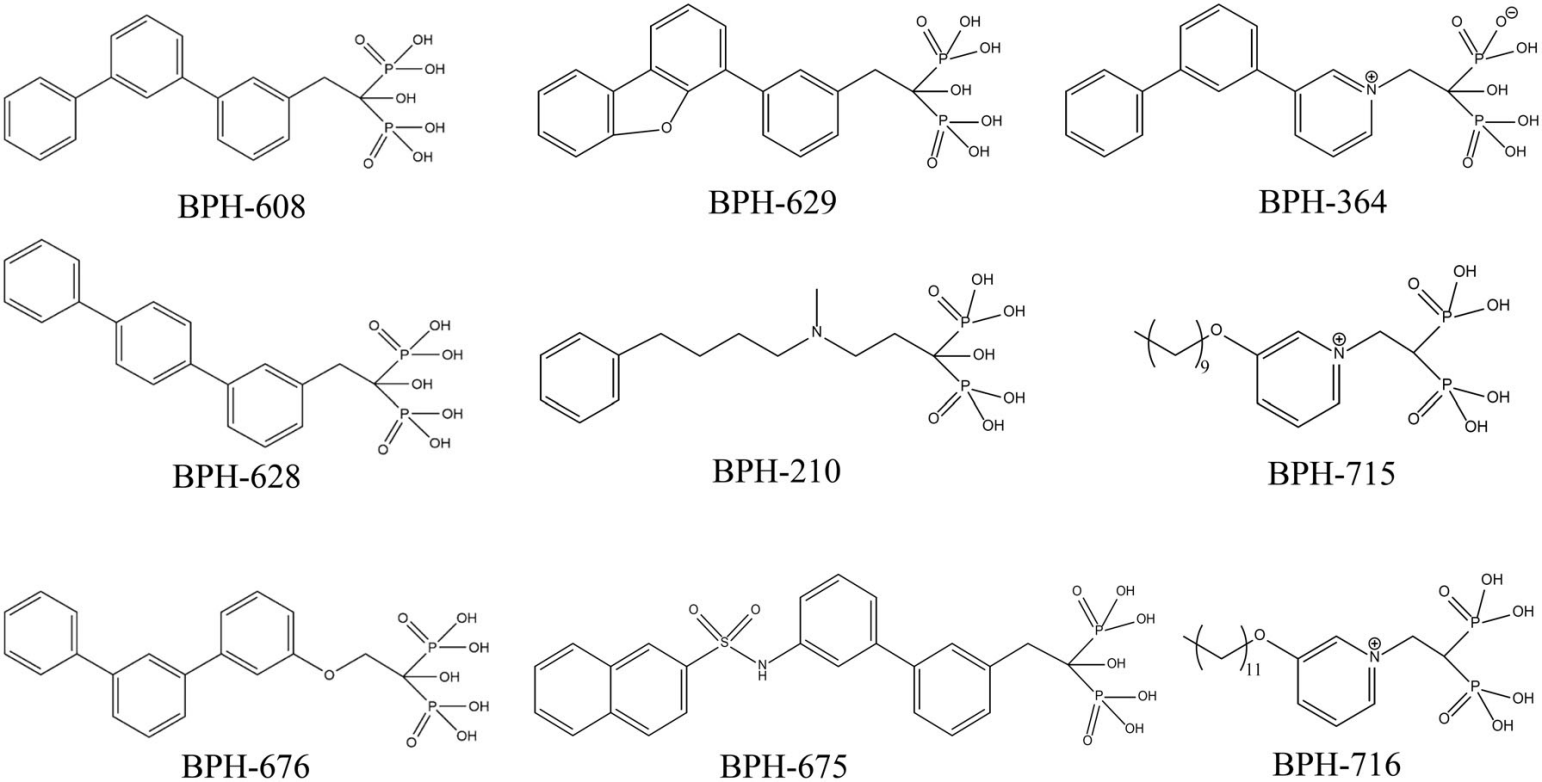
Structures of bisphosphonates investigated as potential head‐to‐tail prenyl synthase inhibitors. BPH‐608, ‐628, ‐629, and ‐676 for bacterial undecaprenyl diphosphate synthase inhibition; BPH‐364, ‐629, ‐675, and ‐210 for yeast geranylgeranyl diphosphate synthase inhibition; BPH‐210 also good for *Trypanosoma brucei* farnesyl diphosphate synthase inhibition; BPH‐715 and ‐716 for tumor cell growth inhibition

Isoprenoids comprise a wide variety of natural products that play roles in cell wall synthesis, plasma membrane construction, electron transfer in the respiratory chain, appropriate cellular localization, and signaling activity in bacteria, fungi, and animals, including humans. Therefore, the steps in isoprenoid biosynthesis and metabolism are valuable targets in discovering new inhibitors or drugs. For instance, bacterial UPPS, which generally serves as an antibacterial target, catalyzes consecutive head‐to‐tail condensation reactions to form 55‐carbon UPP, a lipid carrier for bacterial peptidoglycan biosynthesis.^35^, ^36^, ^37^, ^38^, ^39^, ^40^ Chen et al. used farnesyl thiopyrophosphate (FsPP), an FPP analog, as a probe to detect UPPS inhibition on the basis of conformational changes.^54^ Guo et al. were the first to propose crystal structures of bacterial UPPS in a complex with certain potent bisphosphonate inhibitors, BPH‐608, −628, −629 and − 676, which exhibited the most active inhibition at an IC_50_ of 590 nM (Figure 3).^55^ Kuo et al. solved the crystal structure of *Helicobacter pylori* UPPS and found potent inhibition activities against *H. pylori* UPPS by virtually screening 58,635 compounds, thus indicating the possibility of developing antibiotics specifically targeting pathogenic bacteria rather than other intestinal probiotics.^56^ During bacterial cell wall synthesis, UPP is dephosphorylated to undecaprenyl monophosphate as a precursor of lipid‐I and ‐II by membrane integral undecaprenyl pyrophosphate phosphatase (BacA/UPPP).^57^, ^58^ In a recent breakthrough, the structure and kinetic mechanism of UPP have been determined; thus, it is a promising target for antibiotic development (see Section 8 for details).^59^, ^60^, ^61^, ^62^, ^63^ For drug discovery and design, however, pharmaceutical scientists should consider that human homologous enzymes, such as DHDDS and dolichyl pyrophosphate phosphatase, have similar catalytic reactions to produce approximately 100‐carbon dolichols for protein glycosylation reactions in the ER.^64^

FPPS and GGPPS—the enzymes upstream of farnesyltransferase (FTase) and protein geranylgeranyl transferase (GGTase), respectively—were considered attractive targets for anticancer drugs through the inhibition of subsequent protein Ras or Rab prenylation in cell signaling and survival pathways (see Section 7 for details). Various bisphosphonate inhibitors as cancer chemotherapeutics have been reported to inhibit FPPS and GGPPS. Guo et al. proposed several crystal structures of yeast GGPPS in a complex with potent bisphosphonate inhibitors (BPH‐364, ‐629, and ‐675; Figure 3). Among them, BPH‐364 exhibits the most active inhibition at IC_50_ = 30 nM and K_*i*_ = 10 nM.^55^, ^65^ Another potent phenylalkyl bisphosphonate, N‐[methyl(4‐phenylbutyl)]‐3‐aminopropyl‐1‐hydroxy‐1,1‐bisphosphonate (BPH‐210), against *Trypanosoma brucei* FPPS (IC_50_ of 250 nM and K_*i*_ = 21 nM) and yeast GGPPS has been reported.^66^ Zhang et al. also designed and modified a series of lipophilic bisphosphonate inhibitors with long‐chain hydrophobic carbon tail (BPH‐715 and ‐716; Figure 3), which exhibited high inhibition activity against FPPS and GGPPS probably due to increased cellular uptake.^67^

## 3 CLASS 2: HEAD‐TO‐HEAD PRENYL SYNTHASE

### 3.1 Terpenes or isoprenoids synthesis by head‐to‐head prenyl transferases

**FIGURE 4:**
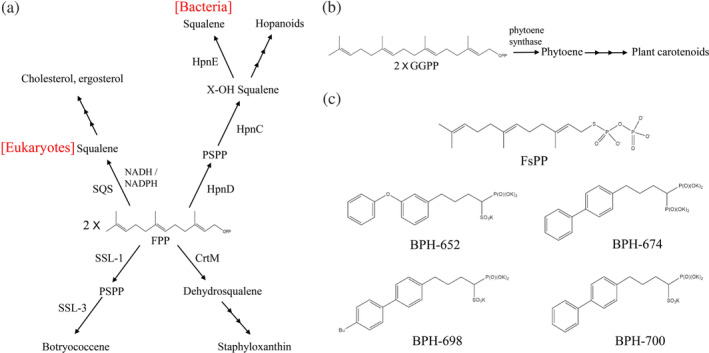
Natural isoprenoids synthesized by head‐to‐head prenyl transferases. (a) Certain crucial head‐to‐head condensation reactions catalyzed by squalene synthase, HpnD, CrtM, and SSL‐1 enzymes in bacteria and eukaryotes. (b) Two geranylgeranyl diphosphate molecules are converted by phytoene synthase to phytoene during plant carotenoid biosynthesis. (c) Structures of bisphosphonates investigated as potential CrtM inhibitors against the virulent pathogen *Staphylococcus aureus*

More than 80,000 identified isoprenoid natural compounds are synthesized using a wide range of prenyl synthases.^1^ Among them, one class, so‐called head‐to‐head prenyl transferases, can be found in microbes, fungi, plants, and animals (including humans). These enzymes, including HpnD,^18^ CrtM,^68^, ^69^ and SQS,^70^, ^71^ mainly catalyze two FPP molecules through a head‐to‐head condensation to form presqualene diphosphate (PSPP), followed by conversion of PSPP to either dehydrosqualene (DHS) or hydroxysqualene (HSQ) and reduction of PSPP to either squalene or botryococcene^18^, ^68^, ^72^ (Figures 4a and S1). These intermediate building blocks are generally involved in the biosynthesis of ergosterol, cholesterol, hopanoids, and staphyloxanthin for maintaining the membrane rigidity and fluidity or act as a virulence factor due to the antioxidant properties in bacteria and eukaryotes (Figure 4a). In plants, two GGPP molecules can be head‐to‐head condensed by phytoene synthases to phytoene, a 40‐carbon intermediate formed in the biosynthesis of plant carotenoids (Figure 4b).^73^ Because these enzymes catalyze the committed step of the synthesis of carotenoid or sterol‐like intermediates and are highly conserved across various species, pharmacologists consider their inhibitors as potential therapeutic leads in the drug development against pathogens and for treating hyperlipoproteinemia.^74^, ^75^, ^76^ Over the past few years, the core mechanism of and structural information on several crucial head‐to‐head prenyl transferases have been well established, which provide advanced insights into inhibitor optimization and drug development for further possible therapeutic applications, including cholesterol‐lowering agents and antimicrobial therapies.^77^, ^78^

### 3.2 Basic building blocks of PSPP biosynthesis in bacteria and eukaryotes

PSPP, a triterpenyl phosphate, is an essential cyclopropyl intermediate in biosynthetic pathways for eukaryotic botryococcene, sterols, bacterial hopanoids, and the carotenoid pigment staphyloxanthin. PSPP can be simply synthesized through the head‐to‐head condensation of two FPP molecules by various enzymes, such as HpnD (involved in HSQ and hopanoid biosynthesis), CrtM (involved in DHS and staphyloxanthin biosynthesis), SQS (involved in squalene and sterols biosynthesis), and SQS‐like enzymes (SSL‐1, involved in botryococcene biosynthesis).^18^, ^68^, ^69^, ^70^, ^71^, ^79^, ^80^ Several elegant studies that conducted crystallographic and biochemical analyses with green algae *Botryococcus braunii* SSL‐1,^79^ *S. cerevisiae* and human SQS,^70^, ^81^, ^82^, ^83^ *Staphylococcus aureus* CrtM,^68^ and *Rhodopseudomonas palustris* and *Zymomonas mobilis* HpnD^18^ have provided detailed mechanisms underlying the joining of the two FPPs in a head‐to‐head coupling to form PSPP, with subsequent reduction and rearrangement to yield other metabolic products.

Liu et al.^13^ determined the crystal structure of CrtM in the virulent pathogen *Sta. aureus* (SaCrtM; DHS synthase), similar to that of human SQS (HsSQS).^17^ Liu et al., therefore, screened numerous SQS inhibitors to inhibit CrtM and thereby block the biosynthesis of staphyloxanthin. Three phosphonosulfonates exhibited a strong inhibition toward CrtM (BPH‐652; K_*i*_ = 1.5 nM; BPH‐698, K_*i*_ = 135 nM; BPH‐700, K_*i*_ = 6 nM), and potent activity against pigment formation of *Sta. aureus* (median inhibitory concentration = 100–300 nM; Figure 4c). BPH‐674 was a more potent CrtM inhibitor (K_*i*_ = 0.2 nM), but with no significant activity in pigment inhibition in vitro, probably because of poor cellular uptake. High‐resolution enzyme structural information in a complex with crucial inhibitors was also proposed. Because staphyloxanthin acts a virulence factor in *Sta. aureus*, inhibiting its production could result in the production of reactive oxygen species from host neutrophils, significantly hindering bacterial growth.^17^ Schwalen et al. also proposed the structural characterization of CrtM and HpnD enzymes from pathogens *Enterococcus hirae* and *Neisseria meningitides*, respectively, thus providing valuable information regarding anti‐infective or antimicrobial drug development against pathogenic bacteria.^68^ A combination of structural information, mutagenesis, and computational modeling can help elucidate the role of Arg‐15 residue (equivalent to Ser‐19 in CrtM) in stabilizing PSPP formation in HpnD. When CrtM Ser‐19 was substituted with arginine, the rate of DHS production decreased, indicating a distinct mechanistic reactivity between staphyloxanthin and hopanoid biogenesis.^68^

### 3.3 Squalene biosynthesis

**FIGURE 5:**
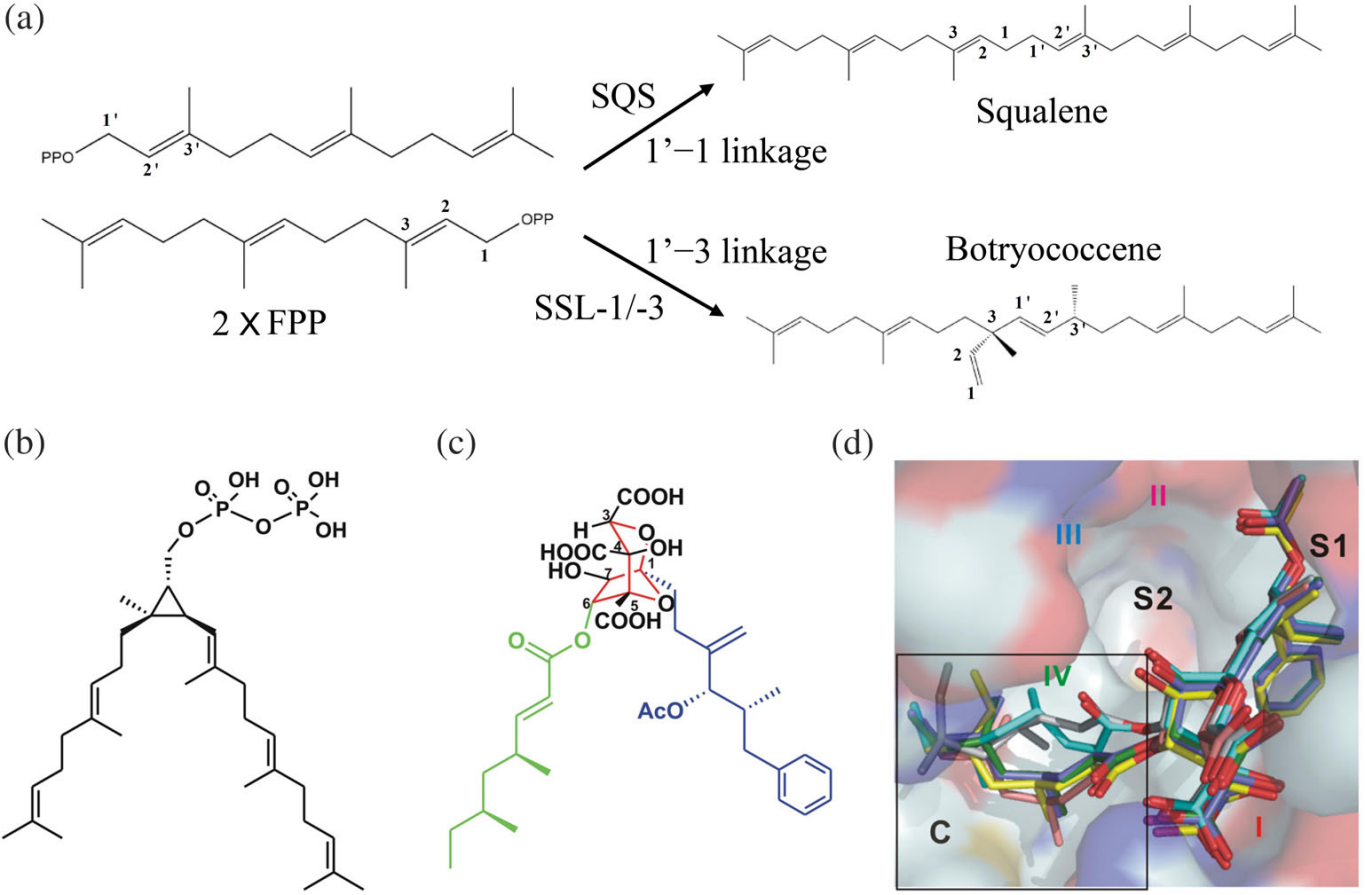
Reactions catalyzed by “head‐to‐head” prenyl transferases. (a) In green algae, photosynthetic *Botryococcus braunii* can catalyze two farnesyl diphosphate molecules to generate squalene via 1′−1 by squalene synthase (SQS) or form botryococcene via 1′−3 linkage by SSL‐1/‐3 enzymes. Potent inhibition of zaragozic acid A (ZA‐A) on human SQS. Structures of presqualene diphosphate (PSPP) (b) and ZA‐A. ZA (c) is a polyketide natural product produced by fungi and shares partial structural similarity with PSPP. (d) Crystal structures of human SQS in complex with ZA‐A molecule shown as stick models. Reprinted (b–d) from our previously published data (Reference 70). Originally published in Reference 70. Copyright 2012 American Society for Biochemistry & Molecular Biology

Squalene is a metabolic intermediate in the biosynthetic pathway for eukaryotic cholesterol and ergosterol and bacterial hopanoids. This naturally occurring terpenoid hydrocarbon is essential in maintaining membrane rigidity and fluidity.^74^ In humans, squalene can be simply synthesized through the head‐to‐head condensation of two FPP molecules by a NADH/NADPH‐dependent SQS.^83^ In other eukaryotes, for example, the photosynthetic green algae *B. braunii* can catalyze two FPPs to form either squalene or botryococcene through 1′−1 or 1′ − 3 linkage by SQS and SSL‐1/‐3, respectively.^79^, ^80^ Botryococcene is a branched triterpene hydrocarbon and is believed to have potential applications in biofuel production (Figure 5a). By contrast, in bacteria, *Z. mobilis* and *R. palustris* have been proposed to produce squalene by using three reactions catalyzed by three enzymes, HpnD, HpnC, and HpnE. This brand new pathway also plays a crucial role in the biosynthesis of hopanoids for enhancing membrane integrity in bacteria.^18^, ^84^

Because SQS catalyzes the committed step of sterol synthesis in bacteria and humans, it has attracted considerable attention as a potential target for generating antimicrobial and anticholesterolemic inhibitors. Through a thermodynamics analysis, Liu et al. demonstrated the inhibitory activity of zaragozic acid A (ZA‐A) on human SQS at nano‐ to picomolar concentrations (*K*_*d*_ = 18.9 nM).^70^ ZA is a polyketide natural product produced by fungi and shares partial structural similarity with PSPP (Figure 5b,c). The molecular mechanism of binding modes of ZA‐A with human SQS suggested that the C‐1 alkyl group of six ZA molecules stays at S1 site, whereas the extensive C site is occupied by the C‐6 acyl group with a distinct binding pattern (Figure 5d). This finding indicates the possibility of developing new drugs and treatments for hypercholesterolemia.

In fungi and some parasitic protozoa, SQS is involved in the ergosterol biosynthesis pathway and was thought to serve as a classic drug target for many infectious diseases. Song et al. recently proposed that ZA (IC_50_ 270 nM), phosphonosulfonate BPH‐652 (IC_50_ 430 nM), and celastrol (IC_50_ 830 nM) have potent inhibition activities against SQS in *Aspergillus flavus* (AfSQS), the leading cause of human invasive aspergillosis.^71^ Celastrol, a natural quinone methide, is a noncompetitive inhibitor of AfSQS and can inhibit the flavin‐dependent monooxygenase siderophore A in the siderophore biosynthesis of the highly virulent *A. fumigatus*, which could serve as a promising multitarget lead for antifungal medication.^71^ In trypanosomatid parasites, SQS has also been proposed as a target for Chagas disease therapeutics because of its essential role in ergosterol biosynthesis. Shang et al. solved the first crystal structure of SQS in *Trypanosoma cruzi* in a complex with certain potent inhibitors of quinuclidine and lipophilic bisphosphonate and demonstrated potent cell growth inhibition on blocking sterol production.^85^

## 4 CLASS 3: HEAD‐TO‐MIDDLE PRENYL SYNTHASE

### 4.1 Mechanisms of action of head‐to‐middle condensation

**FIGURE 6:**
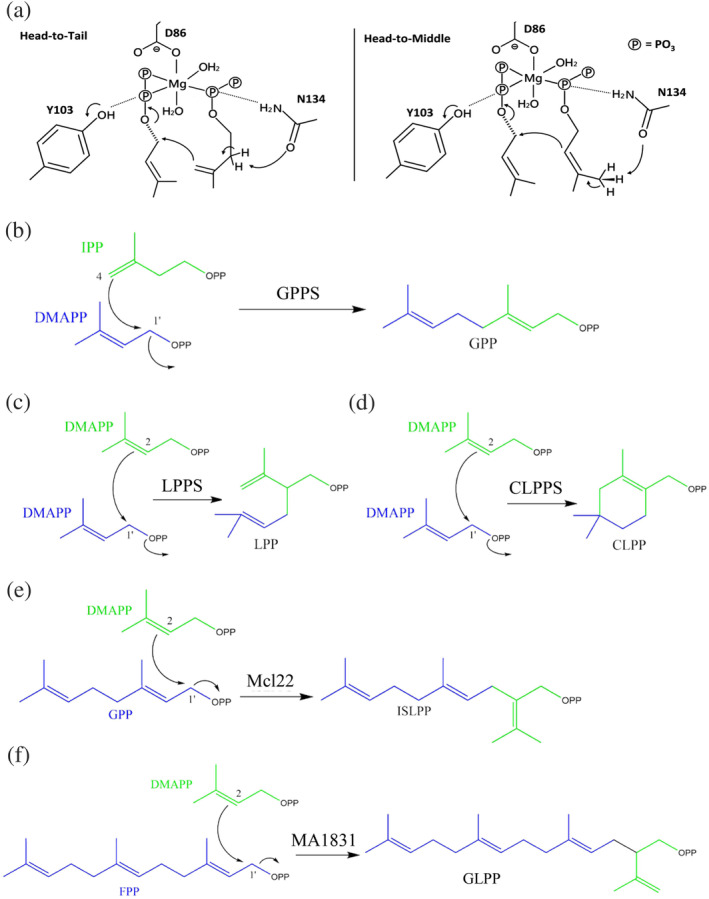
(a) Two proposed mechanisms of the condensation reaction of Z,Z‐farnesyl diphosphate synthase (zFPS) in the tomato species *Solanum habrochaites*; zFPS can catalyze one dimethylallyl diphosphate (DMAPP) and two isopentenyl diphosphate (IPP) molecules to form a Z,Z‐farnesyl diphosphate (Z,Z‐FPP) via the head‐to‐tail condensation (left panel) and two DMAPP molecules to form an LPP via the head‐to‐middle condensation (right panel). Reprinted from our previously published data (Reference 86). Originally published in Reference 86. Copyright 2017 American Chemical Society. ( https://pubs.acs.org/doi/10.1021/acsomega.6b00562; further permissions related to the material excerpted should be directed to the ACS). Reactions catalyzed by “head‐to‐tail” prenyl transferase as shown in geranylgeranyl diphosphate synthase (GPPS) (b) and by “head‐to‐middle” prenyl transferases, as shown in LPPS, CLPPS, Mcl22, and MA1831 (c–f)

Irregular prenyl synthase, a noncanonical type of UPPS‐fold‐like prenyltransferase, was discovered recently. It catalyzes the head‐to‐middle (i.e., non–head‐to‐tail) condensation reaction of the C2–C3 double bond of the first allylic substrate (located at S2 site) and C1 in a second allylic substrate (located at S1 site), subsequently producing branched mono‐ or sesquiterpenes (Figure 6a). Some well‐known examples are 10‐carbon lavandulyl diphosphate (LPP), catalyzed by lavandulyl diphosphate synthase (LPPS) or Z,Z‐farnesyl diphosphate synthase (zFPS)^86^, ^87^; 10‐carbon cyclolavandulyl diphosphate (CLPP), catalyzed by cyclolavandulyl diphosphate synthase (CLPPS)^88^; 15‐carbon isosesquilavandulyl diphosphate (ISLPP), catalyzed by isosesquilavandulyl diphosphate synthase (Mcl22)^89^; and 20‐carbon geranyl lavandulyl diphosphate (GLPP), catalyzed by MA1831, a *cis*‐prenyltransferase homologue from *Methanosarcina acetivorans* (Figure 6b–f).^90^

The crystal structure and molecular mechanism of head‐to‐middle LPPS were first reported by Liu et al. in 2016.^87^ This irregular prenyl synthase catalyzes the coupling reaction of C2 in the first DMAPP (S2 site) and attacks C1 in the second DMAPP allylic substrate (S1 site) to form the 10‐carbon LPP, a precursor of lavandulol, which is used as a lavender oil fragrance in the perfume industry (Figure 6c).^87^ The structural information of its homologous zFPS, with ~39% amino acid sequence identity to LPPS, was proposed by Chan et al. within a similar timeframe. zFPS, discovered from the glandular trichomes of the tomato species *Solanum habrochaites*, catalyzes two DMAPPs to form a LPP molecule if IPP is absent in the reaction.^86^ The overall structure of LPPS is quite similar to those of zFPS and other *cis*‐prenyl diphosphate synthases, such as UPPS. However, the catalytic residue His‐78 in LPPS, which plays a key role in the head‐to‐middle condensation reaction and facilitates the release of diphosphate from the S1 ligand, is replaced with asparagine in UPPS (Asn‐28) and zFPS (Asn‐88). Replacement of His‐78 with an asparagine residue (H78N) did not result in LPP production in LPPS.^86^ However, mutagenesis for the functional analysis of this equivalent residue in either UPPS (Asn‐28) or zFPS (Asn‐88) has not been investigated.

A unique irregular prenyl synthase, CLPPS, catalyzes the head‐to‐middle condensation of two DMAPP molecules, followed by a cyclization reaction to form a branched and cyclic 10‐carbon CLPP (Figure 6d). The molecular mechanism and function of CLPPS have been increasingly studied by organic chemists and biologists because of the ability of CLPPS to consecutively catalyze both condensation and cyclization reactions, which are generally accomplished by two independent enzymes, isoprenyl diphosphate synthase and cyclase (see Section 5 for details), in isoprenoid biosynthesis. Tomita et al. recently solved the crystal structure of CLPPS from the soil bacterium *Streptomyces* sp. CL190 and indicated a high structural similarity to UPPS‐like proteins. Site‐directed mutagenesis (P8I/F173L mutant) combined with GC/MS analyses suggested that a narrower catalytic pocket is suitable for the approach of two dimethyl moieties of DMAPP for cyclization in CLPPS, whereas a wider catalytic pocket is required for maintaining a longer distance between two dimethyl moieties of DMAPP for condensation in LPPS.

Another irregular prenyl synthase, Mcl22, may be involved in the production of the merochlorin, a meroterpenoid antibiotic produced by the marine bacterium *Streptomyces* sp. strain CNH‐189.^91^ This enzyme catalyzes the head‐to‐middle condensation of DMAPP (S2 site) and GPP (S1 site) to generate a branched 15‐carbon ISLPP (Figure 6e). Although the overall polypeptide fold of each domain in Mcl22 is similar to that of *cis*‐prenyl transferases, the width of the ligand‐binding pocket (between helices α2 and α3) compared with ligand‐binding pockets of other UPPS enzymes is narrow.^89^ This spatial restriction allows the binding of the GPP substrate in a horizontal position at the surface of the binding pocket (PBD ID: 5XK9), unlike other UPPS homologs, whereby the substrate binds (such as FPP, citronellyl diphosphate, and tuberculosinyl diphosphate) in a vertical orientation toward the hydrophobic tunnel during catalysis. This unique spatial control for ligand binding and catalysis may prove to be a suitable strategy in engineering new merochlorin‐class antibiotics. Moreover, MA1831, a homologue of Mcl22, was recently demonstrated to produce 20‐carbon GLPP from one DMAPP and FPP in *M. acetivorans* (Figure 6f). However, its structure and catalytic mechanism remain unclear.^90^ Whether MA1831 produces a molecular machinery similar to that in Mcl22 must be determined.

### 4.2 MoeN5 prenyltransferase in moenomycin biosynthesis

**FIGURE 7:**
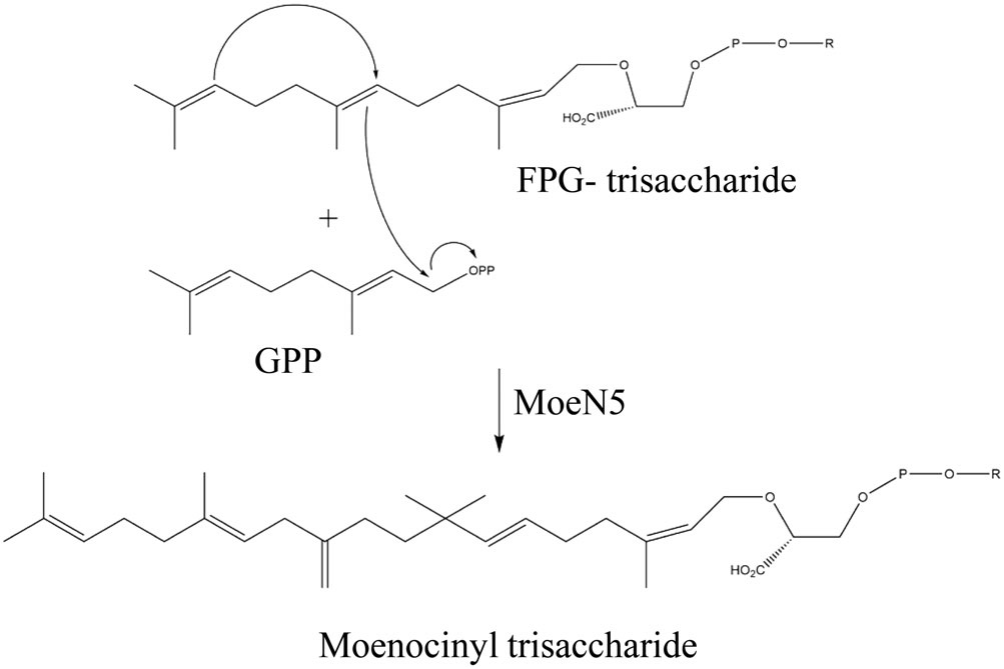
Proposed mechanisms of the head‐to‐middle condensation reaction of MoeN5. R indicates trisaccharide

MoeN5 can catalyze one GPP molecule (S1 site) and the *cis*‐farnesyl group in the phosphoglycolipid (FPG‐trisaccharide; S2 site) through head‐to‐middle condensation to produce the 25‐carbon moenocinyl‐side‐chain‐containing lipid in the biosynthesis of moenomycin (Figure 7). The crystal structure and reaction mechanism of MoeN5 were recently reported by Zhang et al.^20^ MoeN5 forms a dimer both in the solution and in the crystal structure. Unlike the previously mentioned head‐to‐middle enzymes, MoeN5 exhibits a structural similarity to head‐to‐tail *trans*‐prenyl synthases, such as geranyl transferase and GGPPS, which generally contain two canonical aspartate‐rich domains, DDXXD and DXXXD, coordinating Mg^2+^ ion for the ionization of allylic substrates. Crystal structures of several ligand‐bound complexes of MoeN5 provide a structural basis for a rational catalytic reaction mechanism, in which the long side‐chain of FPG‐trisaccharide exhibits a unique bent conformation (C6–C11) to attack the C1 of GPP, followed by the formation of intermediate of 5‐ and 6‐membered‐ring carbocations and then the 25‐carbon moenocinyl side‐chain.^20^

## 5 CLASS 4: TERPENOID CYCLASE

### 5.1 Distinct structures and mechanisms underlying cyclization

Terpene cyclase, a type of prenyltransferase, catalyzes the cyclization of linear isoprenyl pyrophosphates, such as GPP, FPP, and GGPP, into various cyclic isoprenoid compounds. Terpene cyclases include squalene cyclase, pentalenene synthase, 5‐*epi*‐aristolochene synthase, and trichodiene synthase, responsible for the synthesis of cholesterol, a precursor of the pentalenolactone (a sesquiterpenoid antibiotic), the antifungal phytoalexin capsidiol, and antibiotics and mycotoxins, respectively (Scheme 1). These enzymes are generally divided into two groups, mainly characterized by the distinct α‐helical folds and chemical catalysis mechanisms. Group I terpenoid cyclases are metal‐dependent (Mg^2+^ or Mn^2+^) and usually contain two conserved aspartate‐rich DDXX[D/E] and NSE[N/D]DXX[S/T]XX[K/R]E (NSE/DTE) motifs for metal ion binding. These two motifs form a trinuclear metal cluster to initiate the ionization of an isoprenoid diphosphate substrate to an allylic cation, which acts as an electrophile to react with one of the π bonds in the substrate for cyclization. By contrast, group II enzymes contain one DXDD motif in which the central aspartic acid serves as a catalytic general acid for the initiation of protonation of a carbon–carbon double bond in an isoprenoid diphosphate substrate to form a tertiary carbocation for cyclization.^1^, ^92^ The 3D crystal structures of terpenoid cyclases exhibit considerably different α‐helical folds. The crystal structure typically comprises three domains, namely α, β, and γ, in various combinations. For instance, *Taxus brevifolia* taxadiene synthase (group I), which synthesizes Taxol for cancer chemotherapy, consists of α, β, and γ domains.^93^ 5‐*epi*‐aristolochene synthase (Group I) in tobacco, *Nicotiana tabacum*, comprises α and β domains.^94^ Pentalenene synthase (Group I) in bacterial *Streptomyces exfoliatus* contains only one α domain.^95^ Squalene‐hopene cyclase (group II) in bacterial *Alicyclobacillus acidocaldarius* comprises β and γ domains.^96^ In group I cyclases, the catalytic site is located in the middle of the α domain, whereas in group II cyclases such as squalene‐hopene cyclase, the catalytic site is located in the β–γ domain interface.

**FIGURE 8:**
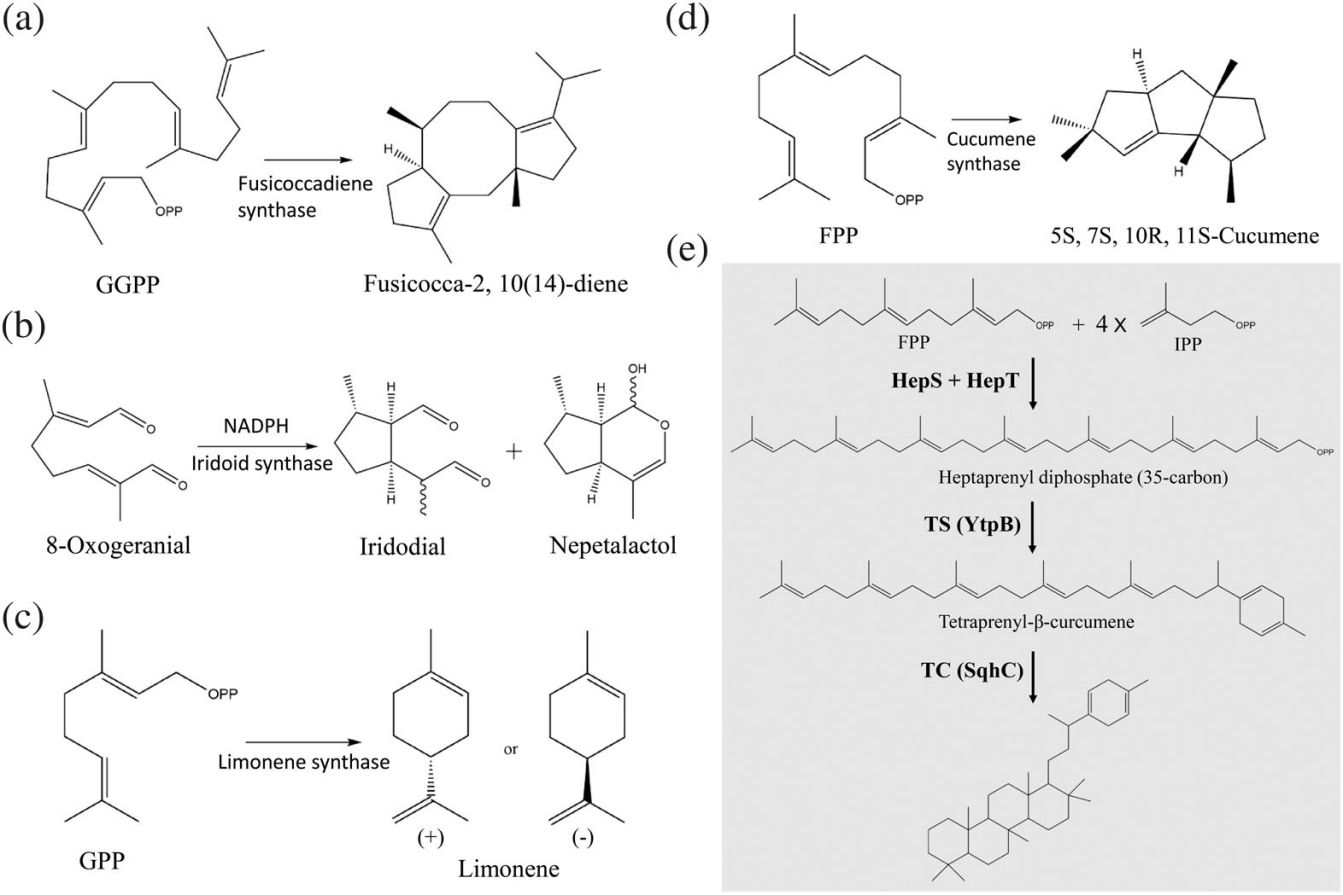
Certain critical cyclization reactions catalyzed by terpene cyclases, as shown in fusicoccadiene synthase (a), iridoid cyclase (b), limonene synthase (c), and cucumene synthase (d). (e) Potential biosynthesis pathway for sesquarterpenes (C35 Terpenes). The heterodimeric heptaprenyl diphosphate synthase (HepS and HepT) converts one farnesyl diphosphate and four isopentenyl diphosphate molecules via a head‐to‐tail condensation reaction to form heptaprenyl diphosphate. This reaction is subsequently followed by cyclization by YtpB and SqhC enzymes, generating one ring at one end of a 35‐carbon chain of heptaprenyl diphosphate and 35‐carbon terpenol with a polycyclic skeleton, respectively. Reproduced from Reference 97. Originally published in Reference 97. Copyright 2011 American Chemical Society

Several elegant studies on crystallographic and functional analyses of certain distinct terpene cyclases have provided detailed mechanisms of how a linear prenyl diphosphate molecule undergoes changes in bonding through ionization and rotation during a multistep cyclization reaction to generate various metabolic compounds containing one or more fused rings (Figure 8a–d). In 2016, Chen et al. solved the crystal structure of fusicoccadiene synthase, which can cyclize GGPP to form molecular fusicoccadiene (Figure 8a), the hydrocarbon precursor of fusicoccin‐A applied in cancer chemotherapy.^98^ Within a similar time frame, Kries et al. determined the crystal structure of plant iridoid cyclase (*Catharanthus roseus*), which can catalyze a different substrate, 8‐oxogeranial, into iridodial and nepetalactol (Figure 8b).^99^ Morehouse et al. subsequently reported the crystal structure of orange limonene synthase, which catalyzes GPP via a simple cyclization reaction to form molecular limonene, which is used as a fruit oil fragrance (Figure 8c).^100^ Finally, Blank et al. also recently solved the crystal structure of cucumene synthase, which can generate a linear triquinane sesquiterpene from molecular FPP, which is useful for its anticancer, antibiotic, and anti‐inflammatory properties (Figure 8d).^101^

### 5.2 New subclass: Sesquarterpene cyclase

Despite the rapid progress in understanding the biological function and synthetic mechanism of a large diverse group of terpenoid natural products, little is known regarding the 35‐carbon terpene. Only 19 cyclic and 8 linear 35‐carbon terpenes have been identified to date. Sato et al. suggested a brand new biosynthesis pathway of mono‐ and pentacyclic C35 terpenes by two unique terpene cyclases, namely tetraprenyl‐β‐curcumene synthase (YtpB) and tetraprenyl‐β‐curcumene cyclase (SqhC), in *B. subtilis* and *Mycobacterium* species.^97^ The mono‐ and pentacyclic terpene compounds are derived from the 35‐carbon heptaprenyl diphosphate, which was synthesized by one FPP and four IPP molecules through head‐to‐tail condensation by heterodimeric enzymes HepS and HepT (Figure 8e).^45^ YtpB can catalyze the ionization and cyclization of heptaprenyl diphosphate to form a ring at one end of the 35‐carbon chain, but with no amino acid sequence homology to any known terpenoid synthase. Moreover, YtpB does not contain the canonical DDXX(D,E) or NSE/DTE metal‐binding motifs typically found in group I terpenoid synthases.^97^ Although the structural information of YtpB in terpene cyclization is currently unknown, Fujihashi et al. recently proposed the crystal structure of BalTS, an ortholog of YtpB from *B. alcalophilus*. Interestingly, the overall structure of BalTS is similar to that of group I terpene synthases, but it consists of two novel aspartate‐rich motifs, DYLDNLxD and DY(F,L,W)IDxxED, which forms the cyclic skeleton.^102^ More recently, Stepanova et al. also determined the crystal structure of BalTS in a complex with a substrate surrogate and elucidated the catalytic mechanisms in unusual large terpene synthesis and in drug design applications. On the basis of these findings, the authors designated 35‐carbon terpene as a new family named sesquarterpenes.^103^

## 6 CLASS 5: AROMATIC PRENYLTRANSFERASE

### 6.1 Classification

Aromatic prenyltransferases catalyze the transfer of isoprenoid diphosphates to aromatic compounds, a reaction corresponding to Friedel–Crafts alkylation. This essential prenylation of aromatic compounds in biological systems generates a wide diversity of primary and secondary metabolites, which play critical roles in bacteria, fungi, and plants. This superfamily is generally divided into three groups, characterized by its secondary metabolisms and localization of biological activities^1^: cytosolic ABBA (α‐β‐β‐α barrel) type,^2^ dimethylallyltryptophan synthase (DMATS) type, and 3) membrane‐embedded UbiA type.^23^, ^104^, ^105^, ^106^

### 6.2 Cytosolic ABBA prenyltransferase

NphB (formerly named Orf2) was the first proposed ABBA‐type prenyltransferase, involved in the biosynthesis of the antioxidant naphterpin in *Streptomyces* sp. (strain CL190).^107^ NphB has been reported to possess high tolerance to aromatic substrates, including resorcinols, flavonoids, and dihydroxynaphthalenes, that interact with GPP to generate various O‐ or C‐prenylated aromatic products. The 3D crystal structure of NphB exhibits a 10‐stranded antiparallel β‐barrel fold and consists of five repetitive αββα elements. Its catalytic site is located in the middle of the β‐barrel fold and contains an aspartate‐rich metal‐binding motif (NDxxD). NphB catalyzes the isoprenyl diphosphate substrate by using the Mg^2+^ ion.^107^ By contrast, some ABBA‐type enzymes, such as CloQ and DMATS, do not require Mg^2+^ to initiate the ionization of prenyl diphosphate substrate.^22^ CloQ, originally isolated from *Str. roseochromogenes*, is involved in the biosynthesis of clorobiocin, an aminocoumarin antibacterial that inhibits DNA gyrase. A primary and tertiary structural analysis revealed that CloQ does not contain the NDxxD motif for metal ion binding and remains active in the absence of any divalent cations. The reaction mechanism may be based on the hydrogen bond interactions for substrate ionization.^22^ DMATS is essential in the biosynthesis of ergot alkaloids, originally isolated from the ergot fungus *Claviceps purpurea*, which are secondary metabolites with a high value in regulatory toxicology and pharmacology.^108^ DMATS shows no amino acid sequence homology to NphB, CloQ, or orthologs but shares a similar conformation of β‐barrel fold with the ABBA‐type superfamily according to a number of solved crystal structures (PDBID: 3I4X; 3O2K; 6OS3).^22^ Recent studies on the crystal structure of the prenyltransferases AmbP1 and AmbP3 have indicated that these prenyltransferases can catalyze the 5‐ or 10‐carbon intermediate prenylation of hapalindoles.^109^, ^110^ AmbP1 catalyzes GPP to either C‐2 or C‐3 of *cis*‐indolylvinyl isonitrile in the presence or absence of Mg^2+^, whereas AmbP3 can transfer DMAPP on C‐2 of the hapalindole compound in a reverse manner. Structural and biochemical data clearly indicated that both enzymes exhibit flexible selectivity in hapalindole biosynthesis, which could expand our understanding of the enzymology for new bioactive compound synthesis.^104^

### 6.3 Membrane‐embedded UbiA prenyltransferase

In contrast to cytosolic ABBA prenyltransferases, enzymes belonging to the UbiA superfamily are integral membrane proteins. They catalyze a key biosynthetic step in the production of lipoquinones, including ubiquinone, menaquinone, vitamin E, heme, and other prenylated aromatic compounds (Scheme 1). These natural products play essential roles in important processes such as oxidative phosphorylation, photosynthesis, and antioxidation in all living organisms. Li reviewed a similarity analysis of the sequence and function of the UbiA prenyltransferase superfamily, including COX10, COQ2, DPPR synthase, Archaeal UibA homologue, DGGGP synthase, ChlorophyII synthase, UbiAD1, and MenA.^105^ Studies on the crystal structure of archaeal UbiA homologs, *Aeropyrum pernix* (ApUbiA) and *Achaeoglobus fulgidus* (AfUbiA), have clearly demonstrated the conformation of two aspartate‐rich motifs, NDXXDXXXD and DXXXD, in complexes with GPP or DMAPP and Mg^2+^ ion. The conformation of the catalytic site is similar to the corresponding metal‐binding motifs of farnesyl diphosphate synthase and group I terpenoid cyclases.^111^ The structural and biochemical analyses provide new insights into the molecular basis of the enzyme specificity for aromatic substrates in membrane bilayers.

## 7 CLASS 6: PROTEIN PRENYLATION

### 7.1 Prenylation of farnesyl and geranylgeranyl isoprenoids

**FIGURE 9:**
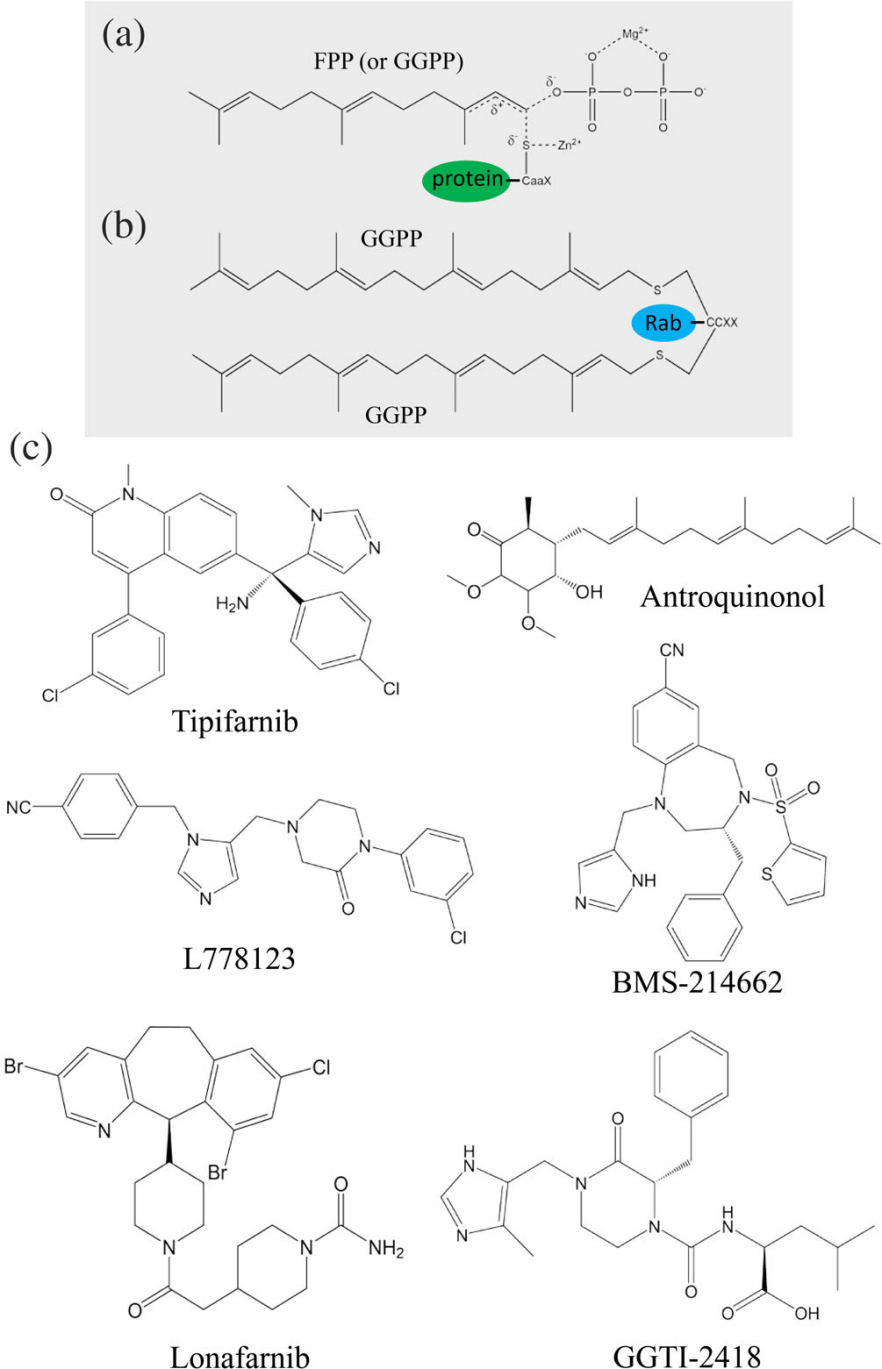
Structures of farnesylated or geranylgeranylated Ras protein and geranylgeranylated Rab protein. (a) FTase or GGTase‐I, a Zn^2+^‐dependent prenyltransferase, catalyzes the transfer of farnesyl diphosphate or geranylgeranyl diphosphate (GGPP) to a conserved cysteine residue in a CaaX motif of Ras protein. (b) Two GGPP molecules can be transferred by GGTase‐II to two conserved cysteine residues in the consensus CCXX region located at the C‐terminus of Rab protein. (c) Structures of six protein prenylation inhibitors investigated in clinical trials

Over the past decades, posttranslational modification has been extensively investigated because of its significance in proper cellular processes and expanding the diversity of protein functions. Posttranslational modification processes, including glycosylation, methylation, acetylation, phosphorylation, and sumoylation, play a crucial role in regulating biological processes such as protein trafficking and cell growth and division in all eukaryotic cells. Protein prenylation, a posttranslational modification, forms an irreversible covalent bond between the protein and either a farnesyl or a geranylgeranyl isoprenoid compound. Three prenyltransferase enzymes are involved in this catalytic reaction: (1) FTase, (2) GGTase type 1 (GGTase‐I), and (3) GGTase type 2 (GGTase‐II or Rab GGTase; Figure 9a,b).^24^, ^112^

Prenylation of Ras protein is initiated by the attachment of a 15‐carbon FPP (by FTase) or a 20‐carbon GPP (by GGTase‐I) to a conserved cysteine residue located at a C‐terminal consensus region commonly known as CaaX box. Next, the –aaX tripeptide of protein C‐terminus is removed by an endoprotease termed Ras‐converting CaaX endopeptidase 1, followed by isoprenylcysteine carboxylmethyltransferase, which catalyzes the methylation reaction of the modified cysteine residue (Figure 9a).^112^, ^113^ However, GGTase‐II is quite different from FTase and GGTase‐I in that it requires an additional Rab escort protein for prenylation activity. GGTase‐II generally catalyzes a single subfamily of Ras‐related small GTPase, which belongs to the Rab protein family involved in the regulation of intracellular vesicular transport in the biosynthetic secretory and exocytic/ endocytic pathways.^114^, ^115^, ^116^ Basically, two GGPP molecules can be transferred by GGTase‐II to two conserved cysteine residues in the consensus region CXC or CCXX located at the C‐terminus of Rab proteins (Figure 9b). Finally, prenylation produces modified proteins with a hydrophobic C‐terminus for further plasma membrane association.

### 7.2 Structural and functional analyses

FTase and GGTase enzymes make crucial contributions to the posttranslational modification of Ras or Rab proteins. FTase, GGTase‐I, and GGTase‐II are Zn^2+^‐dependent prenyltransferases and contain one α and one β domain to form a heterodimer. The primary and tertiary structural analyses revealed that FTase and GGTase‐I share an identical α‐subunit, with a sequence identity of approximately 22% with the α‐subunit of GGTase‐II. By contrast, the β‐subunit of FTase shares only approximately 28% and 32% identity with GGTase‐I and GGTase‐II, respectively.^24^, ^117^, ^118^ The predicted reaction mechanism of prenylation is shown in Figure 9a,b. The Zn^2+^‐activated thiolate of cysteine residue could act as a nucleophile and attack the ionized farnesyl or geranylgeranyl isoprenoid compound. Crystal structures of FTase and GGTase‐I in a complex with FPP, GGPP, or peptide substrates were reported in 2000 and 2003, respectively.^117^, ^119^ Both α and β domains are largely composed of helices, and a Zn^2+^ ion and FPP (or GGPP)‐binding site are located at the center of the barrel surrounded by certain hydrophobic amino acids.^25^ Notably, although the overall structures of FTase and GGTase‐II are quite similar, GGTase‐II has a deeper hydrophobic cavity than FTase for accommodating a longer GGPP isoprenoid.^120^ FPP may serve as a more effective substrate than GGPP even though GGPP binds competitively with FPP to FTase. A rational explanation is that the diphosphate moiety of the 15‐carbon FPP is closer to the catalytic Zn^2+^ ion than that of the 20‐carbon GGPP, which lies within the hydrophobic funnel of FTase. This conclusion also explains the reason that FPP binds to GGTase‐I with 330‐fold lower affinity than GGPP, but still serves as a moderately effective substrate to GGTase‐I during the catalytic reaction.^121^ The Rab prenylation of two GGPP molecules by GGTase‐II is more complex because it involves the participation of the Rab escort protein. The superimposition of the FTase, GGTase‐I, and GGTase‐II structures suggested that GGTase‐II has a more extended binding pocket for the target substrates, and residues in this region are not conserved, reflecting the peptide substrate specificity.^116^, ^122^

### 7.3 Biotechnological and therapeutic applications

Studies have suggested that farnesylation and geranylgeranylation of FTase, GGTase‐I, and GGTase‐II are involved in various clinical conditions such as progeria, infectious diseases, and aging.^123^, ^124^ Among these, cancer remains the leading cause of mortality worldwide.^125^, ^126^, ^127^, ^128^ Therefore, the inhibition of prenylation (prenyltransferase) has provided a new alternative for treating cancer or oncogenic activity. Over the past few years, numerous analogs, including natural isoprenoids, have been synthesized or extracted and subsequently used in investigations into various aspects of the prenylation reaction including its mechanisms, protein structures, enzyme kinetics, and prenylated proteomes. Moreover, some of these inhibitors have been assessed in clinical trials.^24^, ^128^, ^129^, ^130^, ^131^, ^132^ Wu et al. extracted natural terpenoid antroquinonols from *Antrodia camphorate*,^133^ which may inhibit the activity of FTase and GGTase in cancer cells; in turn, this may inhibit the function of Ras and Ras‐related GTP‐binding proteins. Thus far, antroquinonol has been assessed in phase II clinical trials investigating its efficacy against several types of cancer (Figure 9c, Tables 1 and 2).^134^ Another natural cyclic monoterpenoid, limonene, a major component of fruit‐derived essential oils, is a flavoring agent in food manufacturing. Research on its D‐form isomer, *d*‐limonene, has progressed to phase one clinical studies for breast, parotid, and submandibular gland cancers (Figure 8c, Tables 1 and 2).^135^ Other well‐known FTase and GGTase inhibitors with various skeletons, including imidazoles, thiazoles, polycyclics, and pyridines, have been developed and assessed. For example, tipifarnib, lonafarnib, BMS‐214662, L778123, and GGTI‐2418 (PTX‐100) are presently being clinically investigated to determine their roles in treating on various types of tumors and other human diseases including hepatitis, Hutchinson–Gilford syndrome, and neurodegenerative diseases (Figure 9c, Tables 1 and 2).

**TABLE 1:** A summary of clinical trials for protein prenylation inhibitors

Clinical trials			
Compound	Inhibition	Phase	Application in medicine
Tipifarnib	FTase inhibitor	II	Atypical chronic myeloid leukemia, BCR‐ABL1
		II	Chronic myelogenous leukemia, BCR‐ABL1
		I	Adult glioblastoma
		II	Adult acute Monoblastic leukemia
		II	Adult acute myeloid leukemia
		II	Adult acute Monocytic leukemia
		I/II	Untreated childhood brain stem glioma
		II	Anaplastic large cell lymphoma
		II	Estrogen receptor‐positive breast cancer
		II	Recurrent melanoma
		II	Neurofibroma, plexiform
		II	Non‐small cell lung cancer
		II	Bladder cancer
		II	Pancreatic cancer
Antroquinonol	FTase inhibitor	II	Chronic hepatitis B
		I/II	Pancreatic neoplasm
		II	Non‐small cell lung cancer
		II	Atopic dermatitis
		II	Hyperlipidemias
*d*‐limonene	FTase inhibitor	I	Breast cancer
		I	Parotid gland tumor
		I	Submandibular gland tumor
L‐778123	FTase inhibitor	I	Head and neck cancer
		I	Lymphoma
BMS‐214662	FTase inhibitor	I	Myelogenous leukemia
		I	Myelodysplastic syndromes
Lonafarnib	FTase inhibitor	II	Chronic hepatitis D
		II	Hutchinson‐Gilford syndrome (progeria)
		I	Chronic myelogenous leukemia
		I	Brain and central nervous system tumors
		II	Epithelial ovarian cancer
		I	Gliosarcoma
GGTI‐2418	GGTase I inhibitor	I	Advanced cancer
(PTX‐100)			

*Note:* Data obtained from https://clinicaltrials.gov/.

**TABLE 2:** Therapeutic and industrial applications of well‐known natural terpenoid compounds

Natural compound	Source	Application	Reference
Antroquinonol	*Antrodia camphorata*	Chronic hepatitis B, cancer, atopic dermatitis	^a^,^134^
*d*‐limonene	Plant essential oils	Breast cancer, flavoring agent	^a^,^135^
Xanthohumol	*Humulus lupulus*	Metabolic syndrome, oxidative stress	^a^
Squalene	Plants and animals	Virus diseases, respiratory tract diseases	^a^
β‐carotene	Carrot, tomato	Vitamin A source, antioxidant activities	^a^
Fusidic acid	*Fusidium coccineum*	Antibiotic activity	136
Manoalide	*Luffariella variabilis*	Antiinflammatory, antibiotic activity	137
Artemisinin	*Artemisia annua*	Antimalaria	^a^
Coenzyme Q10	Legumes	Inflammation, obesity	^a^
Menthol	*Mentha piperita*	Antibacterial	138
Ingenol mebutate	*Euphorbia peplus*	Actinic keratosis (LEO pharma trade name Picato)	139
Icariin	Epimedium	Osteoporosis, dipolar disorder	^a^
Cannabidiol	*Cannabis sativa*	Analgesic against spasms and asthma	131, 140
Taxol	*Taxis brevifolia*	Cancer therapy	^a^
α‐Santonin	*Artemisia maritima*	Anthelmintic	141
Bisabolol	*Matricaria chamomilla*	Chronic insomnia, cosmetic products	^a^,^142^
Sclareol	*Salvia sclarea*	Fragrant chemical compound, leukemia	143
Carveol	Seeds of caraway	Flavor additive in the food industry	144
Lavandulol	Lavandula	Flavor and fragrance materials	87
Forskolin	*Plectranthus barbatus*	Weight loss	145
Cucurbitacin	*Cucurbitaceae* sp.	Otitis media with effusion	^a^
Moenomycin A	*Streptomyces*	Antibiotic activity	19

^a^ Data obtained from https://clinicaltrials.gov/.

A recent study on crystal structures of FTase from *A. fumigatus*, a human pathogen, made great contributions to the fields of human health and agriculture. Mabanglo et al. proposed that *A. fumigatus* FTase (AfFTase) exhibited some structural differences from the human FTase.^146^ AfFTase exhibited a significant wider substrate binding and product exit groove than that of human; thus, AfFTase provides the potential for developing selective antifungal drugs. The IC_50_ measurement indicated that Tipifarnib (an anticancer agent) preferentially inhibits human FTase, whereas the ethylenediamine scaffold inhibitor ED5 strongly inhibits AfFTase alone. A 3D structural analysis also revealed that AfFTase has a wider binding pocket to accommodate the larger inhibitor (ED5) in the presence of FPP.^146^ However, similar results were not deduced from the crystal structures of human FTase.

## 8 UNDECAPRENYL PYROPHOSPHATE PHOSPHATASE

### 8.1 Bacterial cell wall synthesis

**FIGURE 10:**
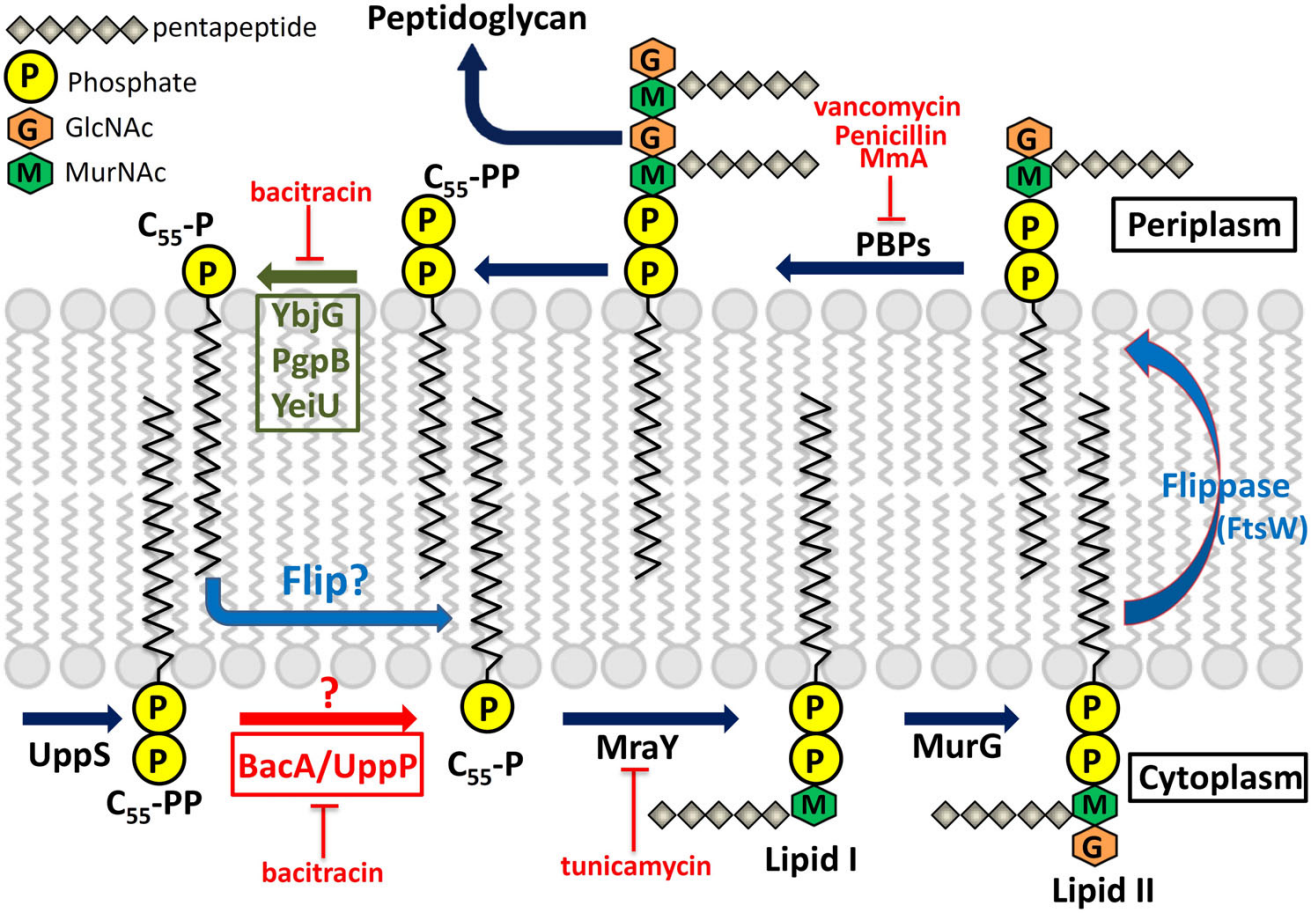
Peptidoglycan biosynthesis in *Escherichia coli*. The undecaprenyl pyrophosphate synthase (UPPS) synthesizes undecaprenyl pyrophosphate (UPP), which is dephosphorylated to undecaprenyl phosphate (UP) by membrane phosphatase BacA/UPPP. The sugar pentapeptides are then transferred to UP by enzymes MraY and MurG for producing lipid I and lipid II, respectively. Lipid II is then translocated to the periplasm via flippase (MurJ, RodA, and FtsW) for peptidoglycan assembly by the penicillin‐binding proteins (PBPs). Finally, UP is flipped back to the cytoplasmic side via an unknown mechanism in the recycling pathway (blue question mark). Certain cell‐wall‐active antibiotics, including bacitracin, tunicamycin, and vancomycin, are also presented. Reprinted from our previously published data (Reference 59). Originally published in Reference 59. Copyright 2014 American Society for Biochemistry & Molecular Biology

The lipid 55‐carbon undecaprenyl pyrophosphate (UPP) is synthesized by UPPS through a consecutive head‐to‐tail condensation reaction of eight IPP and one FPP molecules in the cytoplasm.^32^, ^35^, ^36^, ^37^, ^38^, ^39^, ^40^ Thereafter, UPP is dephosphorylated to undecaprenyl phosphate (UP) by an integral membrane protein, undecaprenyl pyrophosphate phosphatase (BacA/UPPP), through a de novo synthesis pathway (Figure 10).^57^, ^58^ During bacterial cell wall synthesis, UP serves as a carrier lipid for the translocation of the oligosaccharide precursors (lipid II) across the cell membranes via identified flippases (MurJ, RodA, and FtsW) to the periplasm for peptidoglycan assembly.^147^, ^148^, ^149^ Therefore, this essential step in UP metabolism is a potentially viable target when screening for new antibiotics (Figure 10). A recent landmark study determined the crystal structures and kinetics of UPPP, providing valuable insights into the mechanism underlying the enzyme–substrate interaction in membrane bilayers.^59^, ^60^, ^61^, ^62^, ^63^ However, limited information is available regarding the mechanism underlying the flip of UP molecules back to the cytoplasmic site in the recycling pathway. Figure 10 displays the detailed steps involved in polymerization during bacterial peptidoglycan synthesis and summarizes a generally accepted model of carrier lipid biosynthesis in de novo and recycling pathways.

### 8.2 Structural and functional analyses of UPPP

**FIGURE 11:**
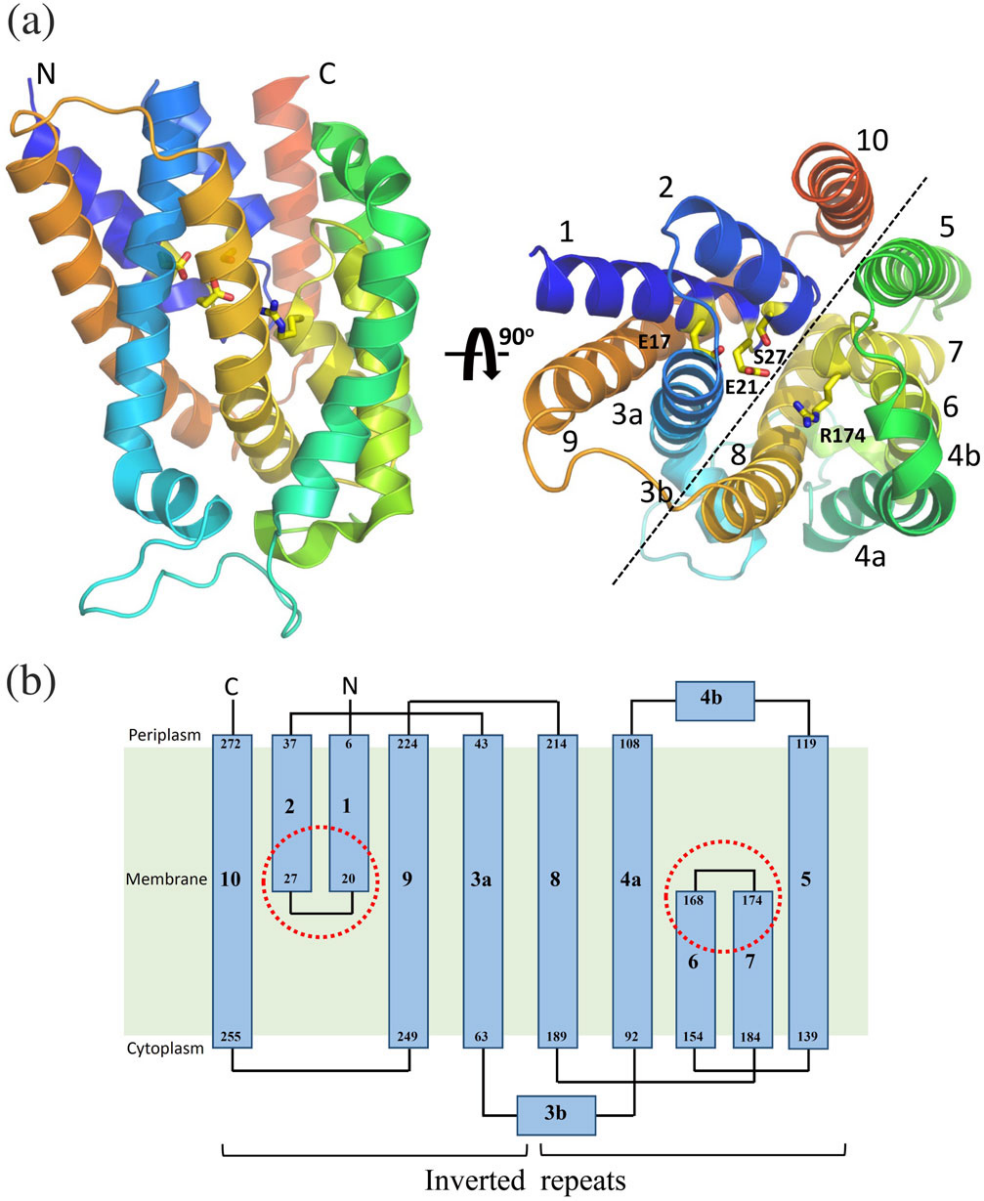
Crystal structure and topology of *Escherichia coli* undecaprenyl pyrophosphate phosphatase (UPPP). (a) Overall structure of *E. coli* UPPP. Structural data are available from the PDB file: 5OON or 6CB2.^62^, ^63^ Ten α‐helices, labeled 1–10, are displayed as a helical cartoon and colored rainbow from the N‐ (blue) to the C‐terminal (red). The orientation of the panel on the right is rotated 90° along the horizontal axis relative to that on the left. The catalytic site residues, Glu‐17, Glu‐21, Ser‐27, and Arg‐174 are presented in yellow and displayed as a stick model. The black dotted line denotes the unique twofold pseudosymmetry axis parallel to the membrane plane. (B) The 10 transmembrane helices are displayed in the two‐dimensional topology of *E. coli* UPPP to highlight the interlocked inverted repeats. The red dotted circles denote two conserved regions (residues 17–30 and residues 170–178) embedded at the membrane midplane

The X‐ray crystal structure of *Escherichia coli* UPPP has been recently reported by Ghachi et al. and Workman et al. in the same time frame.^62^, ^63^ All crystals were obtained through the same method of lipid cubic phase crystallization. UPPP exhibits a dimeric architecture in crystal packing and chemical cross‐linking using disuccinimidyl suberate or succinimidyl succinate. This integral membrane enzyme comprises 10 membrane‐embedded α‐helices, which form two domains with a twofold pseudosymmetrical axis in the plane of the cell membrane (Figure 11a). Six of these are full‐span transmembrane helices (H3–5 and H8–10), and the remaining four form two antiparallel inverted reentrant helices (H1–H2 and H6–H7), which comprise the active site containing two conserved regions (residues 17–30 and residues 170–178) embedded at the membrane midplane (Figure 11b).^62^, ^63^ A topological comparison between UPPP and flippase MurJ reveals that several structural characteristics are shared, including the unique interlocked inverted repeats. This unexpected finding raises the question whether UPPP has any flippase activity during UP recycling back to the cytoplasm. The mechanism underlying these processes remains obscure. Structural and mutagenesis studies have reported that the catalytic event likely begins by serine (Ser‐27) initiating a nucleophilic attack on the phosphorylated center to form a phosphoserine intermediate. Thereafter, a water ion may initiate a second nucleophilic attack on the phosphate ion of the phosphoserine intermediate. Arg‐174 may form a hydrogen bond with the OH group of the pyrophosphate moiety to stabilize the electrophilic phosphocenter during hydrolysis. Glu‐17 and Glu‐21 residues may stabilize the pyrophosphate moiety of UPP through a magnesium or calcium ion, and His‐30 is likely to play a structural role in maintaining stability between H2 and H10 residues.^59^, ^62^, ^63^, ^148^ ^150^

## 9 CONCLUSION

In this review, we compared the molecular structures, catalytic mechanisms, and potent inhibitors of six primary classes of prenyltransferases involved in numerous crucial steps of isoprenoid biosynthesis. The critical role of isoprenoid synthases and the requirement of their exact specificity for distinct catalytic processes indicate a clear mechanistic association between the biogenesis of various natural isoprenoids and normal growth, development, and metabolism of all living organisms. More than 80,000 isoprenoid compounds have been structurally and chemically characterized; however, only a few prenyltransferases have been reported. Therefore, future studies are required to identify new enzyme functions and structures and elucidate the synthetic gene clusters involved in isoprenoid biosynthesis pathways. To identify useful natural products, numerous isoprenoids are of significant commercial value owing to their broad‐spectrum clinical and industrial applications (Tables 1 and 2).^19^, ^128^, ^129^, ^130^, ^131^, ^132^, ^133^, ^134^, ^135^, ^136^, ^137^, ^138^, ^139^, ^140^, ^141^, ^142^, ^143^, ^144^, ^145^, ^150^ At present, the screening, isolation, and identification of new forms of natural compounds with pharmacological properties have gained increasing attention in academia and industry. In recent preclinical and clinical studies, the number of newly identified natural products with therapeutic efficacy in humans against diseases such as cancer, hepatitis, osteoporosis, and respiratory tract diseases has rapidly increased. Moreover, active inhibitors and their derivatives with fewer side effects extracted from natural materials warrant further investigation as a new class of low‐toxicity drugs. These materials may provide novel alternatives for cancer treatment (and treatment of other human diseases) because numerous current therapies for patients with cancers tend to produce strong toxic effects and result in chemotherapeutic resistance. Our results further the current understanding of the formation of these highly diverse carbon‐based materials, which are potentially applicable in the generation of new drugs and food supplements.

## CONFLICT OF INTEREST

The authors declare no conflicts of interest with the contents of this article.

## AUTHOR CONTRIBUTIONS

Hsin‐Yang Chang and Andrew H.‐J. Wang organized and wrote the manuscript. Hsin‐Yang Chang and Tien‐Hsing Cheng designed the tables and figures.

## Supporting information


**Data S1**: Supporting Information
