# Supplementary material for: Structure, catalysis, and inhibition mechanism of prenyltransferase
Source: IUBMB Life. 2020 Nov 27;73(1):40–63. doi: 10.1002/iub.2418 (PMC7839719; doi:10.1002/iub.2418)
Supplement: Supplementary file 1 — Data S1: Supporting Information [file IUB-73-40-s001.docx]

**Table S1. Alphabetical list of abbreviations used throughout the text**

| **Abbreviation** | **Definition** |
| --- | --- |
| ABBA | *α-β-β-α* barrel |
| AtPPPS | polyprenyl pyrophosphate synthase in *Arabidopsis* |
| BacA/UPPP | undecaprenyl pyrophosphate phosphatase |
| CLPP | cyclolavandulyl diphosphate |
| CLPPS | cyclolavandulyl diphosphate synthase |
| DHDDS | dehydrodolichyl diphosphate synthase |
| DHS | dehydrosqualene |
| DMAPP | dimethylallyl diphosphate |
| DMATS | dimethylallyltryptophan synthase |
| DolP | dolichol phosphate |
| FPG-trisaccharide | *cis*-farnesyl group in the phosphoglycolipid |
| FPP | farnesyl diphosphate |
| FPPS | farnesyl diphosphate synthase |
| FsPP | farnesyl thiopyrophosphate |
| FTase | farnesyltransferase |
| GPP | geranyl diphosphate |
| GGPP | geranylgeranyl diphosphate |
| GGPPS | geranylgeranyl diphosphate synthase |
| GGTase | geranylgeranyl transferase |
| GLPP | geranyl lavandulyl diphosphate |
| GPPS | geranyl diphosphate synthase |
| HepS and HspT | heptaprenyl diphosphate synthase |
| HexPPS | trans-hexaprenyl diphosphate synthase |
| HSQ | hydroxysqualene |
| IPP | isopentenyl diphosphate |
| ISLPP | isosesquilavandulyl diphosphate |
| LPP | lavandulyl diphosphate |
| LPPS | lavandulyl diphosphate synthase |
| LSU | large subunit |
| Mcl22 | isosesquilavandulyl diphosphate synthase |
| MEP | methylerythritol phosphate |
| MVA | mevalonate |
| OPP | octaprenyl diphosphate |
| OPPS | octaprenyl pyrophosphate synthase |
| PSPP | presqualene diphosphate |
| SqhC | tetraprenyl-β-curcumene cyclase |
| SQS | squalene synthase |
| SSU | small subunit |
| UP | undecaprenyl phosphate |
| UPP | undecaprenyl pyrophosphate |
| UPPS | undecaprenyl diphosphate synthase |
| YtpB | tetraprenyl-β-curcumene synthase |
| Z,E-DecPP | decaprenyl diphosphate |
| zFPS | Z,Z-farnesyl diphosphate synthase |
|  |  |


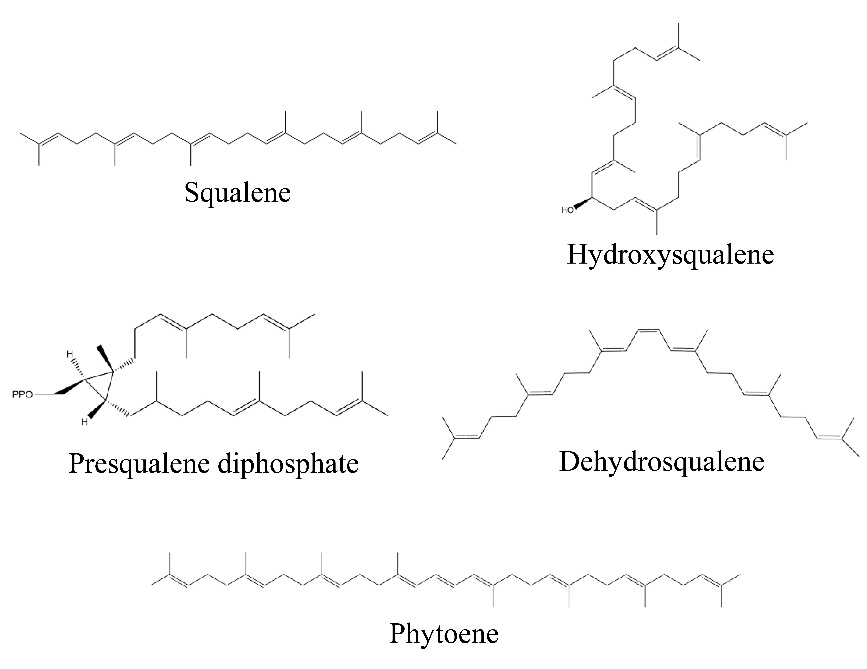


Figure S1. Structures of isoprenoid compounds discussed in the text; OPP indicates diphosphate.
